# Structure and boosting activity of a starch-degrading lytic polysaccharide monooxygenase

**DOI:** 10.1038/ncomms6961

**Published:** 2015-01-22

**Authors:** Leila Lo Leggio, Thomas J. Simmons, Jens-Christian N. Poulsen, Kristian E. H. Frandsen, Glyn R. Hemsworth, Mary A. Stringer, Pernille von Freiesleben, Morten Tovborg, Katja S. Johansen, Leonardo De Maria, Paul V. Harris, Chee-Leong Soong, Paul Dupree, Theodora Tryfona, Nicolas Lenfant, Bernard Henrissat, Gideon J. Davies, Paul H. Walton

**Affiliations:** 1 Department of Chemistry, University of Copenhagen, Universitetsparken 5, 2100 Copenhagen Ø, Denmark; 2 Department of Biochemistry, University Of Cambridge, Tennis Court Road, Cambridge CB2 1QW, UK; 3 Department of Chemistry, University of York, York YO10 5DD, UK; 4 Novozymes A/S, Krogshoejvej 36, 2880 Bagsvaerd, Denmark; 5 Novozymes, Inc., 1445 Drew Avenue, Davis, California 95618, USA; 6 Novozymes North America Inc., 77 Perrys Chapel Church Road, Franklinton, North Carolina 27525, USA; 7 Architecture et Fonction des Macromolécules Biologiques, CNRS, Aix-Marseille Université, 13288 Marseille, France; 8 Department of Biological Sciences, King Abdulaziz University, Jeddah, Saudi Arabia

## Abstract

Lytic polysaccharide monooxygenases (LPMOs) are recently discovered enzymes that oxidatively deconstruct polysaccharides. LPMOs are fundamental in the effective utilization of these substrates by bacteria and fungi; moreover, the enzymes have significant industrial importance. We report here the activity, spectroscopy and three-dimensional structure of a starch-active LPMO, a representative of the new CAZy AA13 family. We demonstrate that these enzymes generate aldonic acid-terminated malto-oligosaccharides from retrograded starch and boost significantly the conversion of this recalcitrant substrate to maltose by β-amylase. The detailed structure of the enzyme’s active site yields insights into the mechanism of action of this important class of enzymes.

## Abstract (web summary)

Lytic polysaccharide monooxygenases (LPMOs) are industrially important enzymes that oxidatively deconstruct polysaccharides. Here, Lo Leggio *et al*. report the activity, spectroscopy and three-dimensional structure of a LPMO of the new CAZy AA13 family active on recalcitrant-retrograded starch.

## Graphical abstract

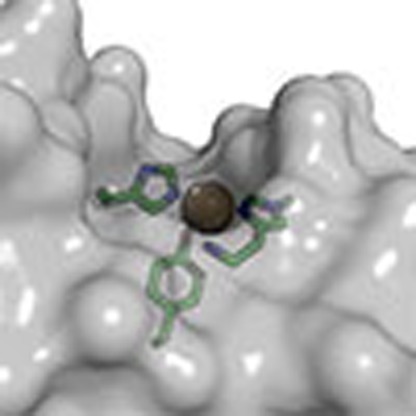

The enzymatic degradation of polysaccharides derived from biomass is essential not only to the global carbon cycle, but also to the fundamental technology that underpins multi-billion dollar industries^1^,^2^. In this regard, the classical model of polysaccharide degradation in which exo-glycosidases operate in synergy with endo-glycoside hydrolases was recently overturned by the discovery of lytic polysaccharide monooxygenases^3^,^4^ (LPMOs, termed PMOs by some), formerly classified as GH61 and CBM33 (ref. 5) enzymes in the CAZy database, but now classified as auxiliary activity (AA) enzymes^6^,^7^. LPMOs utilize molecular oxygen along with plant-derived small molecule, proteinaceous or extrinsic electron sources^4^,^8^,^9^ and have been shown to be copper-dependent monooxygenases^10^,^11^,^12^ that oxidize polysaccharides at C1 (refs 4, 9) and/or C4 (refs 8, 13, 14), thereby instigating chain breakage (hence ‘lytic’) in the polysaccharide. Through oxidative chain breakage, LPMOs thus render the substrate more susceptible to hydrolysis by conventional glycoside hydrolases.

Since their discovery and subsequent characterization in 2010/11, LPMOs have been heralded as a breakthrough in the understanding of fundamental mechanisms that underpin biological biomass utilization. Knowledge of LPMOs has thrown new light on the consortia of enzymes employed by saprophytes in the degradation of recalcitrant polysaccharides. At a molecular level, LPMOs are also challenging convention in metal–oxygen chemistry (reviewed in ref. 15), particularly the detailed biochemistry of the LPMO active site that contains an amino-terminal histidine-ligated mononuclear copper^11^,^12^, termed the histidine brace^3^,^8^,^16^, a structural motif that is also observed in another of nature’s powerful oxygenases, particulate methane monooxygenase^17^. The histidine brace is a strictly conserved feature across all LPMOs that have been structurally characterized, both in the solid state and in solution^11^,^18^,^19^.

LPMOs are becoming key factors in industrial biomass conversion^20^ insofar as they potentially provide access to the full calorific and chemical value of some of the most abundant and recalcitrant polysaccharides, notably cellulose, hemicelluloses and chitin^4^,^9^,^21^,^22^,^23^. Indeed, even though it is only a few years since their discovery, LPMOs are already included in commercial enzyme preparations, which has driven advances in bioethanol production^24^,^25^. Given the fundamental nature of LPMO biochemistry and the major industrial applications of polysaccharide-degrading enzymes, a question is now posed as to the full range of potential substrates on which LPMOs might act, especially starch, which is the most abundant storage glucan in plants and a polysaccharide of industrial importance^26^. Such is the significance of starch-containing crops that their production is greater than for all other industrial or food substrates combined. Not only is starch a major component of the human diet, but is also found in the production of some detergents, high fructose corn syrups, fuel ethanol, textiles, paper and adhesives, with a global market value of ~$51 billion per annum (2012)^27^ (http://www.prweb.com/pdfdownload/10923341.pdf).

The potential for discovery of a starch-active LPMO was hinted at in the influential review by Horn *et al*.^28^ who showed that some LMPOs had associated starch-binding modules, thus signalling that their natural substrate was starch. Indeed, the boosting of starch degradation by enzymes now recognized as starch-active LPMOs on corn starch, amylose and amylopectin was reported in 2010 in the patent literature, where the hydrolysis of these starches by canonical amylo-glucosidases or α-amylases was enhanced by starch-active LPMOs derived from either *Neurospora crassa* (which encodes for a single starch-active LPMO in its genome) or *Aspergillus nidulans*^29^. Very recently, Vu *et al*.^30^ further studied the enhancement by the *N. crassa* LPMO enzyme, *Nc*(AA13), and—commensurate with that expected from the known oxidase activity of LPMOs—showed a reducing agent-dependent oxidative mechanism of action on amylose- and amylopectin-containing substrates. In the same report, solution-state extended X-ray absorption fine structure studies of the active site were suggestive of a histidine brace structure, but the authors were unable to determine whether the copper ion was ligated by two or three histidine residues, raising questions as to whether starch-degrading LPMOs have the same histidine brace structure as observed in all other structurally characterized LPMOs.

It is in this context that we now report the first spectroscopic and full structural characterization of a representative of the starch-degrading LPMO family, hereafter termed AA13. Furthermore, we demonstrate the power of this class of enzymes to render an otherwise recalcitrant form of starch accessible to valorization by classical enzymes, thereby augmenting the range of starch substrates on which AA13 LPMOs have already been shown to be active^29^,^30^. As an exemplar we show that, under certain conditions, β-amylase production of maltose from retrograded starch may be enhanced by up to 100-fold. From structural studies, we also confirm the recent suggestion^30^ that AA13 enzymes have a histidine brace active site, and, as with other fungal LPMOs, feature the N-methylation of N-terminal histidine. We further demonstrate the unique topography of the starch-binding face of AA13s.

## Results

### Genomic identification of starch-degrading LPMO

Using the known starch-binding module CBM20 as an indicator of activity, the novel starch-active LPMO family can be discovered by a ‘module-walking’ approach, akin to that used previously to discover family AA11 (ref. 6). This strategy is based on the fact that carbohydrate-active enzymes are frequently multi-modular proteins in which one or more catalytic domains are appended to carbohydrate-binding proteins (CBMs)^28^. The CBMs provide discrete fingerprints of substrate specificity, traceable through evolution^28^,^31^. Adopting this approach, we found that the CBM20 module, normally associated with GH13 α-amylases, GH15 glucoamylases and other starch-active enzymes, instead appended to a family of proteins (initially called X143, where X-family designates a previously unclassified sequence in the CAZy database) in which the predicted N-terminal residue following signal peptide cleavage is an invariant histidine, leading us to infer that these were potential starch-active LPMOs. The Eijsink group^28^ had also previously noted distant relatives of AA10 LPMOs appended to a starch-binding module.

**Figure 1:**
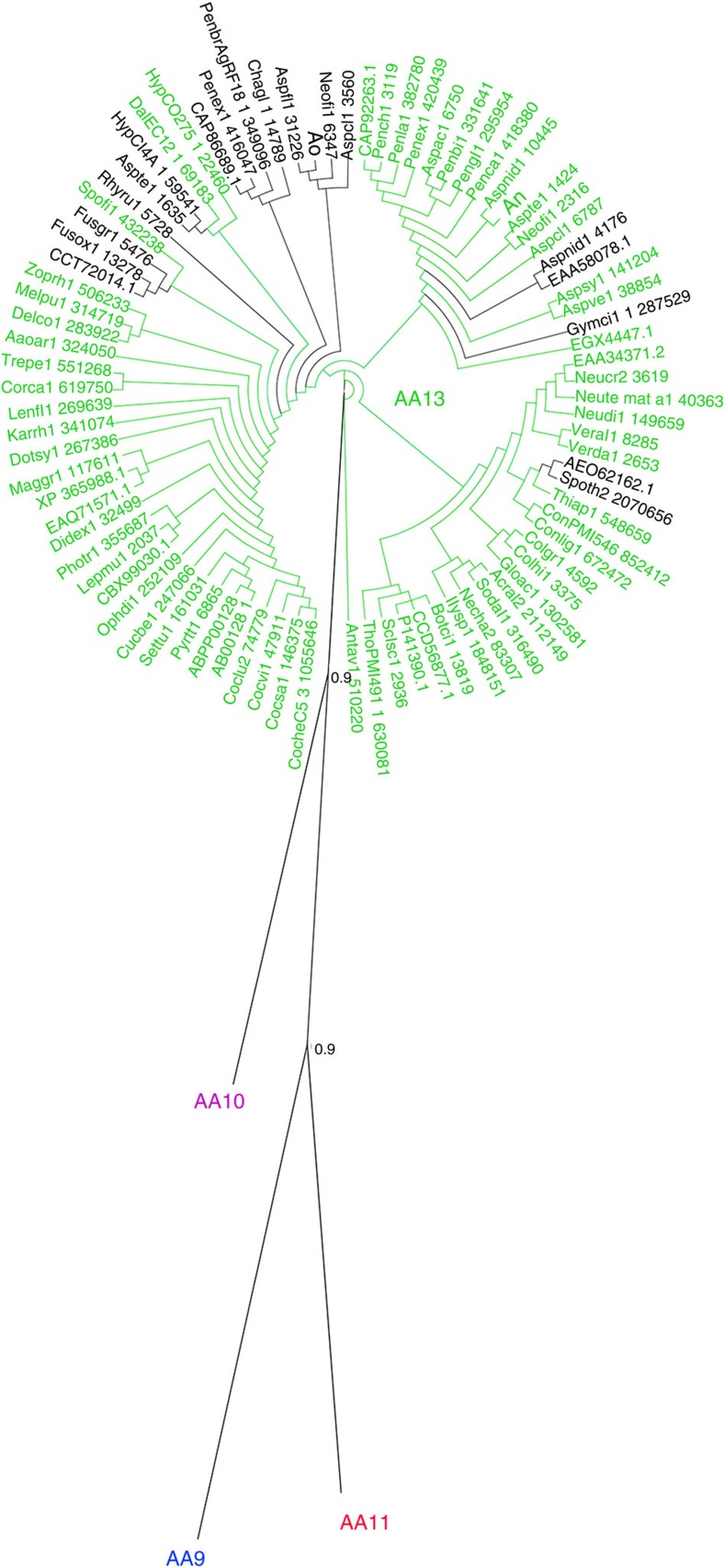
Evolutionary relationship of AA13 with other LPMOs. Composite evolutionary tree with the AA13 part shown as a Newick-like cladogram for clarity and with the other LPMO families (AA9, AA10 and AA11, each with one representative sequence only for clarity) shown with branch lengths that represent phylogenetic distance. Bootstrap values are indicated for the two internal nodes separating the families. The two enzymes studied here are shown by *An* and *Ao* for the *A. nidulans* and the *A. oryzae* AA13 enzyme, respectively. AA13 sequences that are appended to a CBM20 module are shown in green, while those made only of an AA13 module are shown in black. The corresponding circular phylogram is presented in Supplementary Fig. 1.

Pfam analyses conducted with X143 sequences returned only weak scores with Pfam family Chitin_bind_3 (chitin-binding domain, corresponding to the former name of AA10 enzymes, CBM33)^7^. We therefore aligned the X143 sequences, supplemented by several AA9, AA10 and AA11 sequences of enzymes that have been experimentally characterized, and built a corresponding phylogenetic tree (Fig. 1; Supplementary Fig. 1). The tree shows that the X143 sequences strongly cluster together, separate from the other LPMO families with high bootstrap values and thus form a distinct family, which we now designate AA13 (note that class AA12, which received CAZy accession in September 2014 and is redox active, is not an LPMO^32^). To verify the homogeneity of family AA13, a custom-built hidden Markov model was derived from aligned AA13 sequences. This hidden Markov model does not show significant hits to any of the sequences currently classified in CAZy families AA9, AA10 and AA11 and is now routinely being used for the daily updates of family AA13 by the CAZy database (www.cazy.org), thereby—along with *Nc*(AA13) (ref. 30)—defining a new LPMO family in the CAZy database.

The CBM20 modules conjugated to AA13s display strong relatedness (50–70% sequence identity) to many of the (>700 as of 6 November 2014; see http://www.cazy.org/CBM20.html) CBM20 domains that are found appended to GH13 α-amylases and to GH15 glucoamylases (two examples are shown in Supplementary Fig. 2) and for which starch binding has been clearly demonstrated in solution and at the three-dimensional (3D) level. Sequence alignments require no major gaps and show a perfect conservation of the residues conserved in the wider family CBM20, including the known binding residues. We infer from this that the CBM20 appended to AA13 is highly similar to that of CBMs appended to GH13 and GH15, and that its function is starch binding^33^.

The AA13 family currently consists of more than 85 members exclusively found in ascomycete genomes and shows sequence features consistent with LPMO activity, such as two invariant but well-separated histidine residues and aromatic residues associated with a putative electron transfer pathway: Y224, W215, W83, F95 and F161 in *Ao*(AA13). Note that, because the CAZy database only systematically displays entries derived from finished GenBank entries, the corresponding CAZy AA13 page (http://www.cazy.org/AA13.html) currently lists only about 15 of these enzymes, where the remainder belong to formally unfinished genomes. A more complete list of AA13 can thus be obtained by performing a BLAST search of the non-redundant protein database of the NCBI supplemented by a BLAST search of the fungal genomes available from various resources such as the genome portal of the Joint Genome Institute (http://genome.jgi.doe.gov). Inspection of the phylogenetic tree built with 86 sequences for the presence/absence of the appended CBM20 module suggests that the ancestor of family AA13 had a CBM20 and that this domain was occasionally lost in a few lineages.

### AA13 activity on recalcitrant starch

**Figure 2:**
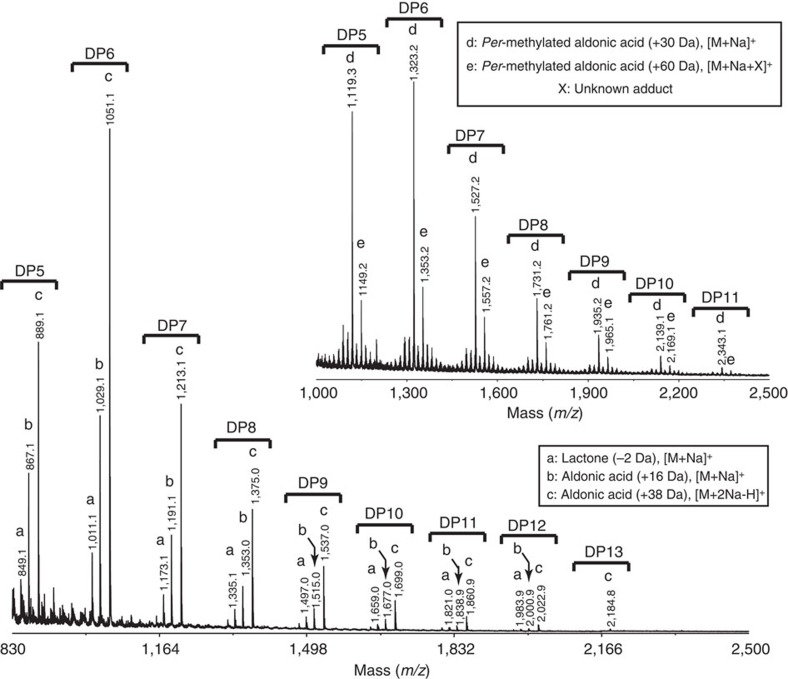
Oxidative breakdown of starch by AA13. MALDI–TOF–MS spectra of *An*(AA13) products from retrograded starch with 4 mM cysteine. C1-oxidized malto-oligosaccharides are present as a monosodiated lactone (*m*/*z* of the malto-oligosaccharide −2 Da), monosodiated aldonic acid (+16 Da) and disodiated aldonic acid (+38 Da). Inset: *per*-methylated C1-oxidized malto-oligosaccharides are present as monosodiated aldonic acid (+30 Da). The mass of unknown adduct X in peak e does not correspond to any cation component of the enzyme preparation.

As an exemplar of the class, an AA13–CBM20 gene from *A. nidulans* was expressed in *A. oryzae* as a secreted protein. The purified *An*(AA13) protein showed moderate activity on retrograded starch, degrading it to aldonic acids dependent on the presence of copper and the reducing cofactor cysteine. This activity was detectable by matrix-assisted laser desorption/ionization–time of flight–mass spectrometry (MALDI–TOF–MS) but not chromatography. MALDI–TOF–MS of the products revealed a series of molecular ions corresponding to modified malto-oligosaccharides with a degree of polymerization (DP) from 5 to 13 (Fig. 2). In contrast to some AA9—AA11 enzymes, unmodified oligosaccharide species were not observed within the detection limits of the instrument. Each oligosaccharide was present as three species: the respective monosodiated or disodiated aldonic acid ([M+Na]^+^, +16 Da relative to unmodified malto-oligosaccharide; [M+2Na-H]^+^, +38 Da) or lactone ([M+Na]^+^, −2 Da). These products are consistent with *An*(AA13) C1-oxidizing activity, as recently reported for *Nc*(AA13) (ref. 30). To confirm this interpretation, the products were *per*-methylated at high pH (thereby opening the lactone to the aldonic acid) and again analysed by MALDI–TOF–MS. The resulting +30 Da species for each DP were consistent with monosodiated *per*-methylated C1-oxidized malto-oligosaccharides (inset Fig. 2). C4 non-reducing end oxidation was not observed, as the +16 Da species that would result from such an action were not detected. To confirm the nature of the oxidation further, the *per*-methylated species were subjected to high-energy collision-induced dissociation MALDI–collision-induced dissociation (Supplementary Fig. 3). Comparison of the spectra of *per*-methylated DP6 malto-oligosaccharide and the corresponding *per*-methylated DP6 AA13 product confirmed that the oxidation occurred at the reducing end. High-performance liquid chromatography analysis of acid-hydrolysed AA13 products further supported the production of gluconic acid (Supplementary Fig. 3c). Together, these data confirm that the reducing end residue of *An*(AA13) products are oxidized at C1. No oxidative activity from AA13 was detected with phosphoric acid-swollen cellulose, chitin, polygalacturonan or esterified pectin, nor with *Arabidopsis* stem cell walls, thus demonstrating a high degree of specificity towards starch-based substrates for *An*(AA13).

**Figure 3:**
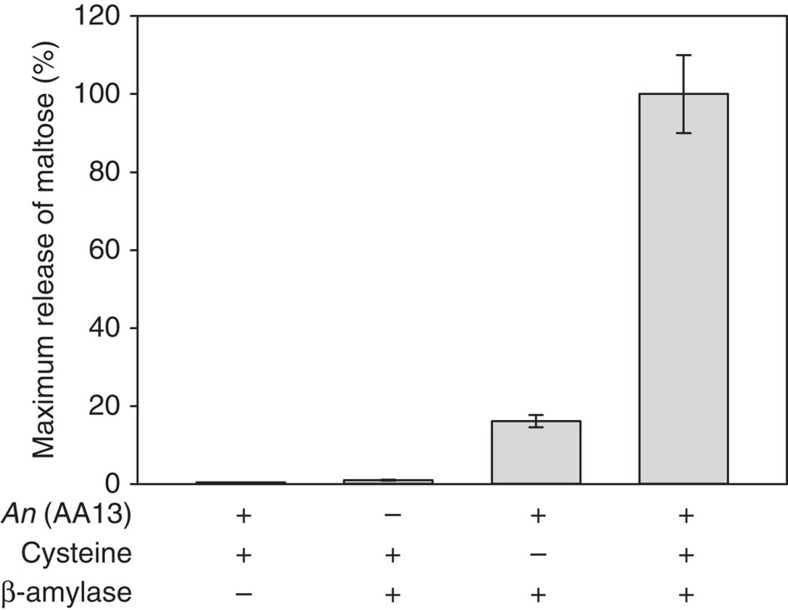
Boosting effect of AA13. Release of maltose from retrograded starch by β-amylase over 4 h at 25 °C (columns 2–4) with *An*(AA13) (column 3), with reducing agent and *An*(AA13) (column 4). Maximum release of maltose was 36.8 nmol, which was 2.5 mol% of starch in the assay. Error bars represent s.e. of triplicate measurements. See Methods for more details.

Notwithstanding the weak activity of *An*(AA13) in releasing oxidized oligosaccharides from retrograded starch, *An*(AA13) was assayed for its ability to act in synergy with a glycoside hydrolase, β-amylase. In the presence of *An*(AA13), large enhancements of maltose release from retrograded starch by β-amylase were observed; the largest enhancements were obtained under specific conditions using the reducing cofactor cysteine, whereby incorporation of *An*(AA13) enhanced the release of maltose by β-*amylase* by *~*100-fold (compared with the same conditions in the absence of the LPMO), consistent with *An*(AA13) acting as an LPMO (Fig. 3). We note the presence of some residual activity from the combination of AA13 and β-amylase in the absence of cysteine as an added reducing cofactor. We attribute this finding to the occurrence of adventitious reducing cofactors present in the starch or β-amylase preparation. Enhancement of maltose release was also observed with a range of different reducing cofactors including ascorbate and pyrogallol, but the greatest enhancement of activity was observed with sulfur-based reductants (Supplementary Fig. 4).

### 3D structure of AA13

To provide structural insight into the AA13 fold, the AA13 gene was expressed from *A. oryzae*, which encodes a protein naturally devoid of a CBM. The structure of this domain (GI:317149073) was solved using the single-wavelength anomalous dispersion method. *Ao*(AA13) activity was tested on solubilized and retrograded starch, chitin and phosphoric acid swollen cellulose (PASC), but no oxidized products were found. We initially thought that the domain might be active on shorter soluble substrates, reflecting the absence of a CBM20 domain, but the enzyme was also inactive on malto-octaose. We can only speculate that lack of a CBM brings the activity below the detection limit of our current methods, see above, or that a partner electron transfer enzyme is required to complete the binding of *Ao*(AA13) to its substrate^30^. Notwithstanding the lack of activity, *Ao*(AA13) is likely to have a very similar structure to that of *An*(AA13) and also that of *Nc*(AA13). This is illustrated by the homology models shown in Supplementary Fig. 5 and supported by the ungapped sequence identity between the two enzymes at 72% (Supplementary Fig. 6d). Furthermore, the copper binding and the subsequent metal-ion spectroscopy for the *An* and *Ao* enzymes are essentially identical, see below.

**Figure 4:**
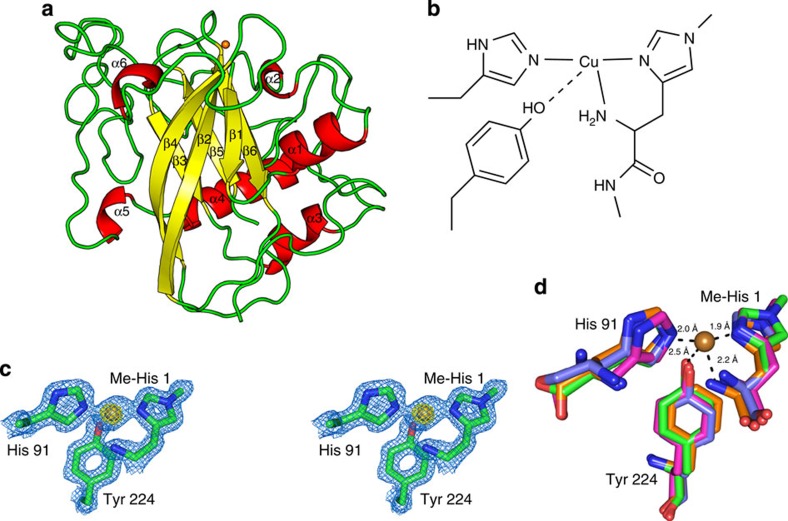
Structural aspects of AA13. Structure of *Ao*(AA13). (**a**) Ribbon view of overall structure with numbered secondary structure elements, copper ion shown as orange sphere; (**b**) line diagram of active site; (**c**) stereo view of the electron density map around the active site in blue (contoured at 1.5σ) with anomalous difference density in yellow (contoured at 25σ), note methylation of N-terminal histidine (the map is calculated from the final refined structure with data collected at a wavelength of 1.037 Å); (**d**) comparison of active site of AA13 (green) with AA9 member (Protein Data Bank (PDB) 3ZUD, magenta, r.m.s.d. for protein atoms shown of 0.73 Å), AA10 member (PDB 2YOY, orange, r.m.s.d. of 0.53 Å) and AA11 member (PDB 4MAI, purple, r.m.s.d. of 0.60 Å).

The refined 1.5 Å Cu-loaded structure of *Ao*(AA13) (Methods; Table 1) has the same overall topology as AA9, AA10 and AA11 LPMOs featuring a conserved central β-sandwich core (Fig. 4a; Supplementary Fig. 7; Supplementary Discussion). Superposition with the available LPMO structures, from the other LPMO families, shows a minimum of 110 aligned residues with a maximum 3.0 Å root mean squared deviation (r.m.s.d.) for Cα atoms with just 10% sequence identity. The active site of AA13 is similar to those found in other characterized LPMOs. Copper coordination is provided by the terminal NH_2_ (Cu-N, 2.2 Å) and the π-N of the side chain of the N-terminal histidine (Cu-N, 1.9 Å), which is again observed to be τ-N methylated, similar to other fungal LPMOs expressed in filamentous hosts^3^,^34^. The endogenous coordination sphere is completed by the τ-N of a further histidine side chain (His91, Cu-N 2.0 Å) and a tyrosine (Tyr224, Cu-O 2.5 Å; Fig. 4b,c), both of which are invariant across the AA13 sequence family. Comparison of the active site residues of AA13 members with those of other LPMOs shows high levels of structural overlap with all atom r.m.s.d. of the two His and Tyr (AA9, AA11, AA13)/Phe (AA10) in the range of 0.52–1.72 Å (Supplementary Table 1; Fig. 4d); there is slightly more positional variation of the adjacent tyrosine group (for AA9 and AA11) with a commensurate variation in the Cu…O distance (2.5–3.2 Å). The copper ion appears to be in the photoreduced copper(I) state, as previously reported for an AA10 member of *Bacillus amyloliquefaciens*^15^ and for an AA10 member of *Enterococcus faecalis*^35^.

**Figure 5:**
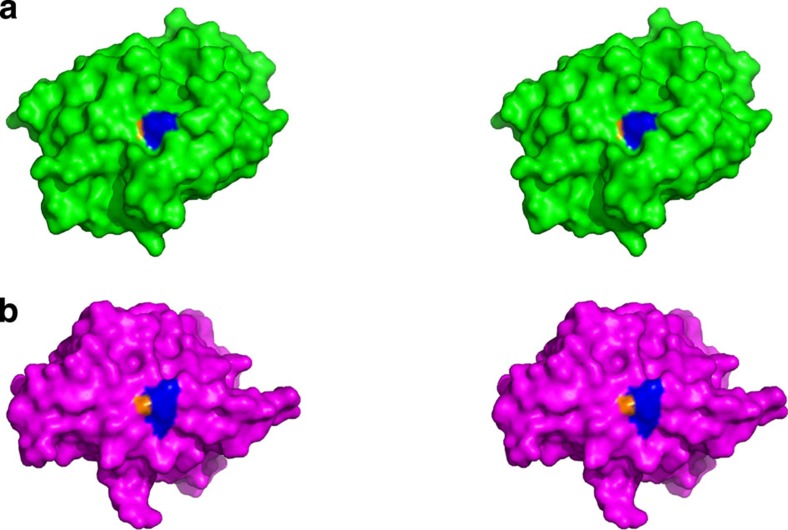
Comparison of substrate-binding faces. Stereo view (divergent, ‘wall-eyed’) of the putative substrate-binding faces of (**a**) *Ao*(AA13) depicting shallow groove leading through copper active site (N-terminal histidine shown in blue, copper ion shown in brown) and (**b**) a cellulose-active LPMO *Ta*(AA9) (Protein Data Bank code 3ZUD) with much flatter binding surface.

As observed in the structures of AA9–AA11, the copper active site in AA13 is presented to solution at the centre of an extended protein face that is presumed to be the surface through which all LPMOs interact with the polysaccharide substrate. In contrast to the flatter binding surfaces seen in AA9–AA11 enzymes, *Ao*(AA13) displays a shallow groove along the protein surface that leads directly through the copper active site. This depression likely accommodates better the profile of a polysaccharide chain within the more contoured surface of a retrograded starch^36^ or possibly a single glucan chain (see Supplementary Discussion), as compared with the flat crystalline surfaces of β-linked polysaccharides with which LPMOs AA9–AA11 interact (Fig. 5). The structural elements of the surface groove of *Ao*(AA13) are derived from the residues in the regions in the long loop preceding β2, the loop between β2 and β3, the long loop preceding β4 and the loop between β5 and β6 (Supplementary Fig. 6). It is not possible to be more definitive about the nature of the AA13–starch interface due to the complexity of the starch structure and the presence of a starch-binding module in most AA13 enzymes that is likely to be the principal determinant of the energetics of the starch–protein interaction. In a final comparison of AA13 with AA9–AA11, it has been proposed previously that AA9 (ref. 8), AA10 (ref. 15) and AA11 (ref. 6) enzymes possess electron transfer pathways from the distal surface of the enzyme through to the active site; the core of *Ao*(AA13) also possesses conserved aromatic groups and cysteine residues that form a close-packed chain consistent with such a role (Supplementary Fig. 10).

### Copper active site properties and spectroscopy

Both *An* and *Ao* AA13 enzymes were subject to metal-site characterization. Isothermal titration calorimetry shows near identical binding of Cu for both with *K*_d_ values of 26±5.2 and 13±8 nM, respectively (Supplementary Fig. 11). The near-axial coordination geometry of the active site copper is reflected in the X-band electron spin resonance (EPR) spectrum of Cu(II)-*An*(AA13) at pH 5, which is essentially identical for both *A. nidulans* and *A*. *oryzae* confirming that the *Ao* 3D structure is a good model for *An*(AA13) (Supplementary Fig. 12). Both spectra exhibit a mononuclear copper-based signal where the semi-occupied molecular orbital (SOMO) has d(*x*^2^−*y*^2^) character along with some rhombicity in the *g*_*x*_ and *g*_*y*_ values. *g*_*z*_ and |*A*_*z*_| values of 2.26 and 162 G (17.1 mK), respectively, place the active site copper within the type 2 classification as defined by Peisach and Blumberg^37^. The EPR spectrum is distinctive from that of other LPMOs insofar as a resolved super-hyperfine coupling to nitrogen ligands (36 MHz, 12.9 G, 1.2 mK) can be observed in the perpendicular region (Supplementary Tables 2 and 3).^6^,^15^,^38^ Whether this reflects an enhanced covalency of the copper-nitrogen bonds in the SOMO or a structurally well-ordered active site structure in AA13 remains to be determined.

## Discussion

This work widens the activity of the AA13 class of LPMOs. *An*(AA13) is the first LPMO to show activity on a recalcitrant ‘retrograded’ starch, predominantly releasing aldonic acid malto-oligosaccharides through lytic oxidative action at the anomeric C1 carbon. Moreover, under specific conditions, the *An*(AA13) enzyme boosts the β-amylase-catalysed release of maltose by up to 100-fold. The 3D structure of AA13 shows that it shares the same histidine brace active-site structure as previous LPMOs and that the extra conserved histidine, commented on by Vu *et al*.^30^ in their solution phase studies, is not part of the copper’s coordination sphere in the solid state structure described here. The structure also reveals that the active site sits within a shallow groove, quite different from the binding surfaces of other known LPMOs. LPMO enzymes have undoubtedly been a revelation, in terms of their unique mononuclear copper chemistry, their impact on industrial process and our understanding of biological oxidative mechanisms. Their diversity, hinted at by their modular structures^6^,^28^, is yet to be fully defined and it is likely that new LPMOs acting on a range of different substrates will yet be identified. Here the analysis of evolutionary markers reflected in appended carbohydrate-binding modules provided the basis for the investigation of a starch-active LPMO family. This approach has potential to reveal new LPMO families, the distinctive metal chemistry and oxidative action of which are set to push the boundaries of biotechnological processes for societal benefit.

## Methods

### Cloning, expression and purification of Ao(AA13)

Cloning and expression of *Ao*(AA13) have been described in US patent application US 2011/0283421, examples 20, 22 and 23 therein. For production of *Ao*(AA13) for purification, *Aspergillus* transformant MStr212 was cultured in YP+2%G medium in baffled flasks shaken at 275 r.p.m. at 30 °C. The culture broth was harvested after 3 days and separated from cellular material by passage through a stack of filters with 1.6, 1.2 and 0.7 μm pore sizes, followed by passage through a 0.45-μm filter. The sterile filtered broth was adjusted to 1.5 M ammonium sulfate and pH 7.5. The broth was purified on a Butyl-650 Toyopearl column (Tosoh Bioscience) equilibrated with 20 mM Tris, 1.5 M ammonium sulfate, pH 7.5 and washed in the same buffer. The enzyme was eluted with a linear ammonium sulfate gradient to 20 mM Tris, pH 7.5. Fractions containing *Ao*(AA13) were combined, concentrated and washed with milli-Q water to a conductivity below 2 mSi cm^−1^ using 10 kDa cutoff Vivaspin 20 (Sartorius). After adjusting to pH 8.0, the enzyme was applied to a Q-Sepharose High Performance column (GE Healthcare) equilibrated with 20 mM Tris, pH 8.0 and washed with the same buffer. The enzyme was eluted with a gradient to 20 mM Tris, 1 M sodium chloride, pH 8.0. Fractions containing *Ao*(AA13) were combined and concentrated as before and purified on a Superdex 75 (26/60) column (GE Healthcare) with a mobile phase of 20 mM MES, 125 mM NaCl solution, pH 6.0. The purified enzyme was buffer changed to 50 mM sodium acetate, pH 5.5 using 10 kDa cutoff Vivaspin 20. For crystallization, 10 mg enzyme in 7 ml was deglycosylated with 0.1 U EndoH (New England Biolabs) for 2 h at 37 °C. The enzyme was cleaned by size exclusion on a Superdex 75 (26/60) column with a mobile phase of 20 mM MES, 125 mM sodium chloride, pH 6.0 and concentrated using 10 kDa cutoff Vivaspin 20.

### Cloning, expression and purification of An(AA13)

Cloning and expression of *An*(AA13) have been described in US patent application US 2011/0283421, examples 1, 5, 6 and 7 therein. The cultivation supernatant was 0.22 μm filtered. The filtrate was adjusted to pH 7.5 and 0.22 μm filtered. The filtrate was brought to 1.5 M ammonium sulfate and left overnight to end precipitation of non-proteinaceous contaminants. The filtrate was subsequently loaded onto a Phenyl Sepharose 6 Fast Flow (high sub) (GE Healthcare, Piscataway, NJ, USA) column XK 26/200 (GE Healthcare, Piscataway, NJ, USA). The column volume (CV) was 70 ml. The column was equilibrated in buffer A (25 mM HEPES, 1.5 M ammonium sulfate, pH 7.5). Unbound protein was washed off with three CVs of buffer A. The proteins were eluted in two steps: (1) a linear gradient: 0–80% of buffer B (25 mM HEPES, pH 7.5) over three CVs and (2) three CVs of 100% buffer B. Fractions were analysed by SDS–PAGE, and fractions containing the enzyme were combined (Supplementary Fig. 13).

The combined fractions were applied onto a gel filtration Sephadex G-25 (GE Healthcare, Piscataway, NJ, USA) column KRONLAB 50/500 (YMC Europe GMBH, Dinslaken, Germany). The CV was 645 ml. The column was equilibrated in buffer A (12.5 mM HEPES, pH 7.5). Fractions containing *An*(AA13) were combined. The combined fractions were applied onto an anion exchange SOURCE 15Q (GE Healthcare, Piscataway, NJ, USA) column KRONLAB 15/125 (YMC Europe GMBH, Dinslaken, Germany). The CV was 20 ml. The column was equilibrated in buffer A (12.5 mM HEPES, pH 7.5). Unbound protein was washed off with two CVs of buffer A. *An*(AA13) was eluted in two steps: (1) a linear gradient: 0–50% of buffer B (1 M sodium chloride in buffer A) over 10 CVs and (2) 3 CVs of 100% buffer B. Fractions were analysed by SDS–PAGE, and fractions containing *An*(AA13) were combined. The combined fractions were applied onto a size-exclusion HiLoad 26/600 Superdex 75 prep grade column (GE Healthcare, Piscataway, NJ, USA). The CV was 320 ml. The column was equilibrated in buffer A (20 mM MES, 125 mM sodium chloride, pH 6). Fractions were analysed by SDS–PAGE, and fractions containing *An*(AA13) were combined.

### Crystallization

Crystals of *Ao*(AA13) could be grown by the vapour diffusion technique in hanging or sitting drop (96-well or VDX plates) using 20% PEG 3000, 0.1 M Buffer system II (ref. 39), pH 5.0 and 0.2 M Zn-acetate as reservoir. Crystals grew spontaneously as plates or single crystals, but could be improved by seeding. Seeding was carried out by transferring a 100-times diluted seed stock with a horse hair in drops consisting of 3 μl protein solution (3 mg ml^−1^) and 1 μl of reservoir. To obtain Cu-loaded crystals, crystals were grown by microseed matrix seeding^40^, with seed stock made in a solution devoid of Zn-acetate and protein stock preincubated with 1 mM copper(II) acetate for 1 h before a new screening round. Crystals grew with a reservoir containing 0.14 M calcium chloride, 0.07 M sodium acetate, pH 4.6, 14% *v/v* isopropanol and 30% *v/v* glycerol.

### Structure determination

A crystal grown without preincubation with copper (II) acetate was soaked in mother liquor containing about 10 mM trimethyl lead acetate for 2.5 h and then cryocooled in liquid nitrogen after mounting on a Hampton Research cryoloop without additional cryoprotection. Crystal information and merging statistics after processing with X-ray diffraction spectroscopy^41^ are shown in Table 1. Despite the suboptimal wavelength (1.008 Å), single-wavelength anomalous dispersion phasing with 36 sites (modelled as Pb) and an initial figure of merit (FOM) of 0.51, followed by density modification in PHENIX^42^ led to an easily interpretable map, where the model could easily be built manually in COOT^43^ and further refined using the CCP4 suite^44^ to an *R*-value of 19.3% and an *R* free of 21.3% with one enzyme molecule per asymmetric unit. Many of the heavy-atom sites turned out later to be Zn rather than trimethyl lead, with disorder at many of the sites. Thus, refinement was continued against data collected for a crystal not soaked with trimethyl lead acetate, but where the protein had been preincubated in the presence of Cu(II) acetate before microseeding, as described above, although the resolution is slightly lower (1.5 Å). Data for refinement were collected at a wavelength of 1.037 Å at beamline 911-2 of MAXLAB, Lund, Sweden. An X-ray fluorescence scan and an additional data set near the copper edge (1.377 Å) were obtained at beamline 911-3 of MAXLAB, Lund, Sweden. The X-ray fluorescence scan clearly showed the presence of all divalent metal ions that were present in the protein buffer or crystallization conditions prior or after seeding, that is, calcium, zinc and copper. Anomalous Fourier maps generated with phases from models where metals were omitted were inspected. The highest peak in the anomalous Fourier map was of the active site metal with a height of 16σ and 47σ at 1.037 and 1.377 Å data collection wavelengths, respectively, confirming its identity as copper. Four additional Zn ions were modelled based on anomalous signal and coordination distances. The active site Cu appears to be mostly in a photoreduced Cu(I) state similar to the structure of an AA10 member from *B. amyloliquefacens*, but with the additional tyrosine ligand also found in AA9 members. However, a small amount of the metal is probably in a Cu(II) state as some residual density that could correspond to a partially occupied water molecule in equatorial position is present (not modelled). No density is observed in apical position.

The final cycles of refinement were carried out with mixed anisotropic/isotropic refinement where protein and associated active site metal were refined anisotropically, while all other atoms were refined isotropically. The final *R* factor was 12.1% and the *R* free 17.0%. The model was deposited at the Protein Data Bank with code 4OPB.

The final electron density has generally very good quality, but revealed a loop spanning the residues Gly165-Arg171 with poor density that was modelled in two conformations. Another residue Ile211 was also modelled with alternative conformations. The active site metal was modelled with occupancy of 1.00, while the additional metal ions possessed occupancies ranging from 0.95 to 0.60. Several molecules present in the crystallization mixture were also modelled, as was the NAG at the glycosylation site at Asn 207. Note that this glycosylation site is not conserved beyond very close homologues of *Ao*(AA13). Two hundred and twelve residues (96.8%) were in the preferred regions of the Ramachandran plot, 6 (2.7%) in the allowed regions and 1 (0.5%) in the disallowed regions, as calculated in COOT^43^. Refinement and validation statistics are given in Table 1. Structures shown are visualized with PYMOL^45^. Superposition of different structures was carried out with the LSQOP program of the CCP4 suite^44^ or COOT^43^. Sequence alignments were made with ClustalX^46^ and STRAP^47^.

### Homology modelling

Homology models of *An*(AA13) and *Nc*(AA13) were prepared with the crystal structure of *Ao*(AA13) as template. Individual residues were mutated in PYMOL^45^ and rotamers were chosen so that clashes were minimized. Subsequently, the structure underwent a geometry analysis in COOT^43^, during which a few residues, presenting critical values, were manually regularized. The structure was minimized in REFMAC5 (ref. 44) by running 10 cycles of structure idealization and finally validated in PROCHECK in CCP4^44^, showing positive G-factors (indicative of good geometry) and no bad clashes. Ramachandran statistics for the *An*(AA13 model) were reasonable with 98.5% of residues in the allowed regions (88.5% in the core regions) and the only four outliers in the flexible loop, which has double conformation in *Ao*(AA13). Most substitutions are conservative, while others, at first sight more radical, for example, a Ser to Arg substitution at position 212 in the core of the protein, can easily be explained by a newly formed salt bridge with Glu125. Ramachandran statistics for the *Nc*(AA13) model were also good with 99.5% of residues in allowed regions (90.4% in the core regions). The only outliers are in a surface-exposed loop. Most substitutions are conservative, while those that are a less trivial are located at the protein surface (for example, Pro172Arg). The homology models are shown in Supplementary Fig. 5, and demonstrate that the *An*(AA13) and *Nc*(AA13) sequence can be accommodated on the structure of *Ao*(AA13) with very few structural changes.

### Measurements of LPMO activity

To ensure that *An*(AA13) was free of any adventitious copper or other metal ions, it was treated as follows. Ten mM ethylenediaminetetraacetic acid (EDTA) was added to demetallate a sample of *An*(AA13). EDTA was then removed by desalting on Sephadex (GE Healthcare, Piscataway, NJ, USA) column KRONLAB 50/500 (YMC Europe GMBH, Dinslaken, Germany). The column was equilibrated in buffer A (25 mM Tris, pH 8.5). Fractions containing the demetallated protein were combined. Copper(II) sulfate (Sigma, C1297) was added to reach 2 × mole equivalents of *An*(AA13) present in the sample. The sample was gently mixed for 15 min. To separate the *An*(AA13) from excess copper(II) sulfate (Sigma, C1297), the mixture was applied to a Sephadex G-25 (GE Healthcare, Piscataway, NJ, USA) column KRONLAB 50/500 (YMC Europe GMBH, Dinslaken, Germany). Retrograded starch was prepared by multiple cycles of freezing and thawing (85 °C, 75 min) of gelatinized corn starch. *An*(AA13) activity was assessed by incubating 100 μl reaction mixtures, containing 0.5% (*w/v*) retrograded corn starch, 100 mM ammonium acetate, pH 6.0, ±4 mM L-cysteine and ±105 pmol *An*(AA13), at 25 °C for 4 h. Reaction mixtures were then boiled for 5 min before 100 μl ethanol was added. The resulting suspension was vortexed and centrifuged, and the supernatant was recovered and dried *in vacuo*. *An*(AA13) products were analysed by MALDI–TOF–MS. *Per*-methylation of oligosaccharides and MALDI–TOF–MS and MS/MS were performed with a 4700 Proteomics Analyzer (Applied Biosystems) using a 2,5-dihydroxybenzoic acid (DHB) matrix^48^,^49^. *An*(AA13) products were separately analysed by High-Performance Anion-Exchange Chromatography Coupled with Pulsed Electrochemical Detection (HPAEC-PAD) following hydrolysis with trifluoroacetic acid^50^.

For β-amylase stimulation assays, a β-amylase solution was prepared by centrifuging 100 μl of a commercial barley β-amylase suspension (Megazyme, Ireland), rejecting the supernatant, then redissolving the pellet in 400 μl 25 mM ammonium acetate, pH 6.0; fractions were then diluted 1,000-fold for stimulation assays. Hundred μl reaction mixtures, containing 0.5% (*w/v*) retrograded corn starch, 100 mM ammonium acetate, pH 6.0, ±4 mM reductant, ±105 pmol *An*(AA13) and ±10 μl 1,000-fold-diluted β-amylase, were incubated at room temperature with constant agitation for 4 h. Reaction mixtures were then boiled for 5 min before 100 μl ethanol was added. The resulting suspension was vortexed and centrifuged, and the supernatant was recovered and dried *in vacuo*. Dried material was then redissolved in 20 μl 50% (*v/v*) dimethylsulphoxide, 7.5% (*v/v*) acetic acid, 6.75% (*w/v*) sodium cyanoborohydride and incubated overnight at 37 °C. The solution was then dried *in vacuo* and redissolved in 100 μl 6 M urea, 4 μl of which was analysed by PACE^51^,^52^.

### EPR spectroscopy

Continuous-wave X-band frozen solution EPR spectra of single sample of 0.2–0.5 mM solutions of CBM20-linked Cu(II)-*An*(AA13) and with 1,000-fold excess of sodium azide (10% *v/v* glycerol) at pH 5.0 (acetate buffer) and 150 K were acquired on a Bruker EMX spectrometer operating at ~9.30 GHz, with a modulation amplitude of 4 G and microwave power of 5.02 mW. Spectral simulation was carried out using Easyspin 4.0.0. Simulation parameters are given in Supplementary Table 2. *g*_*z*_ and |*A*_*z*_| values were determined accurately from the three absorptions at low field. It was assumed that *g* and *A* tensors were axially coincident. Accurate determination of the *g*_*x*_*, g*_*y*_, |*A*_*x*_| and |*A*_*y*_| was not possible due to the second-order nature of the perpendicular region, although it was noted that satisfactory simulation could only be achieved with one particular set of *g* values. At pH 8.5, there is evidence of a second copper species; this second species disappears on the addition of azide, commensurate with partial deprotonation of a copper-bound water molecule at pH 8 and subsequent substitution by azide of the H_2_O/OH from the copper’s coordination sphere. In common with the EPR spectra of other LPMOs, the addition of excess azide is accompanied by a slight change in the *g*_*z*_ value to 2.235 and |*A*_*z*_| value to 173 G, 18 mK.

### Isothermal titration calorimetry

Copper binding was monitored for both *An*(AA13) and *Ao*(AA13) using an auto-ITC200 (GE Healthcare) calorimeter at 25 °C. Protein was demetallated before the experiment with 10 mM EDTA, which was removed by passing the sample down a 16/60 Superdex 75 column equilibrated in 20 mM MES, pH 6, 200 mM NaCl. This buffer had been treated with 5 g l^−1^ of Chelex resin (Sigma Aldrich) for 2 days beforehand to remove any copper that might be present in the solution. The demetallated protein was present in the cell between 40 and 50 μM, with a solution of 500 μM CuCl_2_ present in the syringe prepared in the same buffer. 37 1 μl injections were performed with a 3-min interval between each one. Data were fitted by nonlinear regression using a single-site model in the Origin 7 software package.

## Additional information

**How to cite this article:** Lo Leggio L. *et al*. Structure and boosting activity of a starch-degrading lytic polysaccharide monooxygenase. *Nat. Commun.* 6:5961 doi: 10.1038/ncomms6961 (2015).

**Accession codes:** The structural factor have been deposited with the protein data bank under accession number 4OBP.

## Supplementary Material

Supplementary Information Supplementary Figures 1-13, Supplementary Tables 1-3, Supplementary Discussion, and Supplementary References

## Figures and Tables

**Table 1:** Data collection and refinement statistics.

	**Trimethyl lead acetate soak**	**Cu-loaded crystal refinement data**	**Cu-loaded crystal Cu edge data set**
*Data collection*			
Space group	*P*2_1_2_1_2_1_	*P*2_1_2_1_2_1_	*P*2_1_2_1_2_1_
Cell dimensions			
*a*, *b*, *c* (Å)	47.14, 61.69, 73.83	46.56, 61.60, 73.16	46.57, 61.72, 73.49
*α*, *β*, *γ* (°)	90.00, 90.00, 90.00	90.00, 90.00, 90.00	90.00, 90.00, 90.00
Wavelength (Å)	1.008	1.037	1.377
Resolution (Å)*	30–1.25 (1.32–1.25)	30–1.50 (1.59–1.50)	30–1.70 (1.75–1.70)
*R*_rim_	7.7 (22.6)	8.8 (55.3)	10.2 (70.4)
*I*/*σI*	18.1 (6.3)	13.7 (3.2)	14.3 (3.3)
Completeness (%)	97.2 (84.6)	99.2 (98.7)	99.9% (100.0)
Redundancy	6.8 (4.5)	4.46 (4.42)	6.5 (6.4)
			
*Refinement*			
Resolution (Å)		30.00–1.50 (1.54–1.50)	
No. of reflections		32,929	
*R*_factor_/*R*_free_		12.1/17.0	
No. of atoms			
Protein (incl. Cu)		1,876	
Ligand/ion		62	
Water		216	
B-factors (Å^2^)			
Protein (incl. Cu)		14	
Ligand/ion		36	
Water		27	
R.m.s. deviations			
Bond lengths (Å)		0.019	
Bond angles (°)		1.99	

incl, including; r.m.s., root mean squared.

^*^Values in parentheses are for highest-resolution shell.
