# Supplementary material for: Structure and boosting activity of a starch-degrading lytic polysaccharide monooxygenase
Source: Nat Commun. 2015 Jan 22;6:5961. doi: 10.1038/ncomms6961 (PMC4338556; doi:10.1038/ncomms6961)
Supplement: Supplementary Information — Supplementary Figures 1-13, Supplementary Tables 1-3, Supplementary Discussion, and Supplementary References [file ncomms6961-s1.pdf]

Supplementary Figures

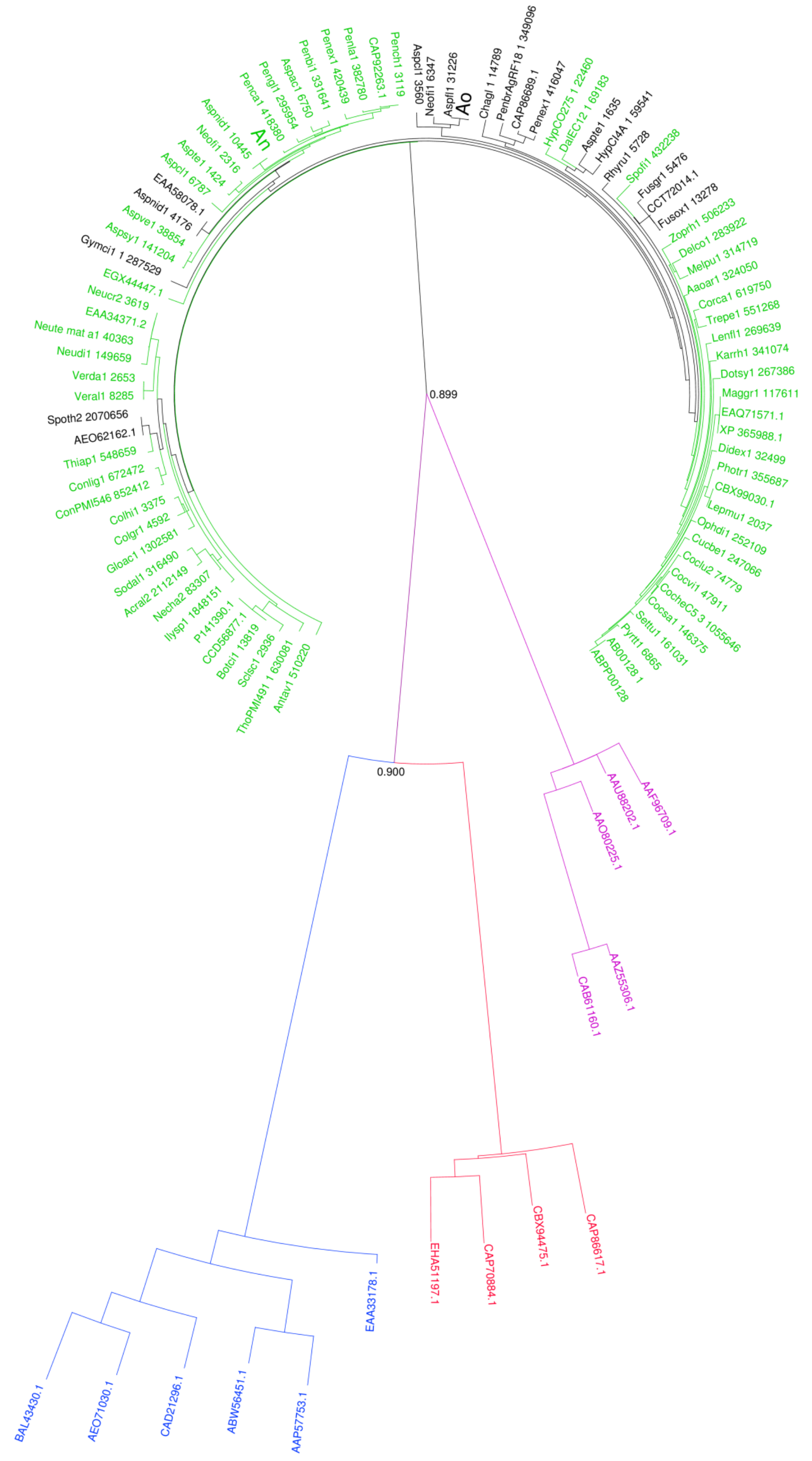

Supplementary Figure 1. Unrooted circular phylogram showing the distinction between the various families of LPMOs. Bootstrap values are indicated for the two internal nodes separating the families. The two enzymes studied here are shown as *An* and *Ao* for the *A. nidulans* (GenBank accession: EAA62623) and the *A. oryzae* (GenBank accession BAE61982.1) AA13 enzymes, respectively. AA9 enzymes are shown in blue, AA11 in red, AA10 in purple and AA13 in green for those that are appended to a CBM20 module and in black for those made only of an AA13 module.

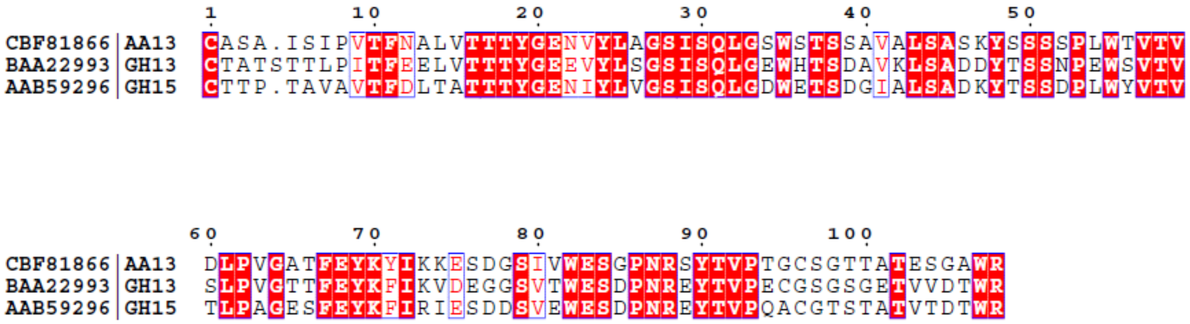

Supplementary Figure 2

Alignment of the CBM20 of *An*(AA13) (top) with examples of known starch-binding domains from GH13 (middle) and GH15 (bottom) enzymes described in PMID=19682075. The GenBank accessions are indicated.

3a)

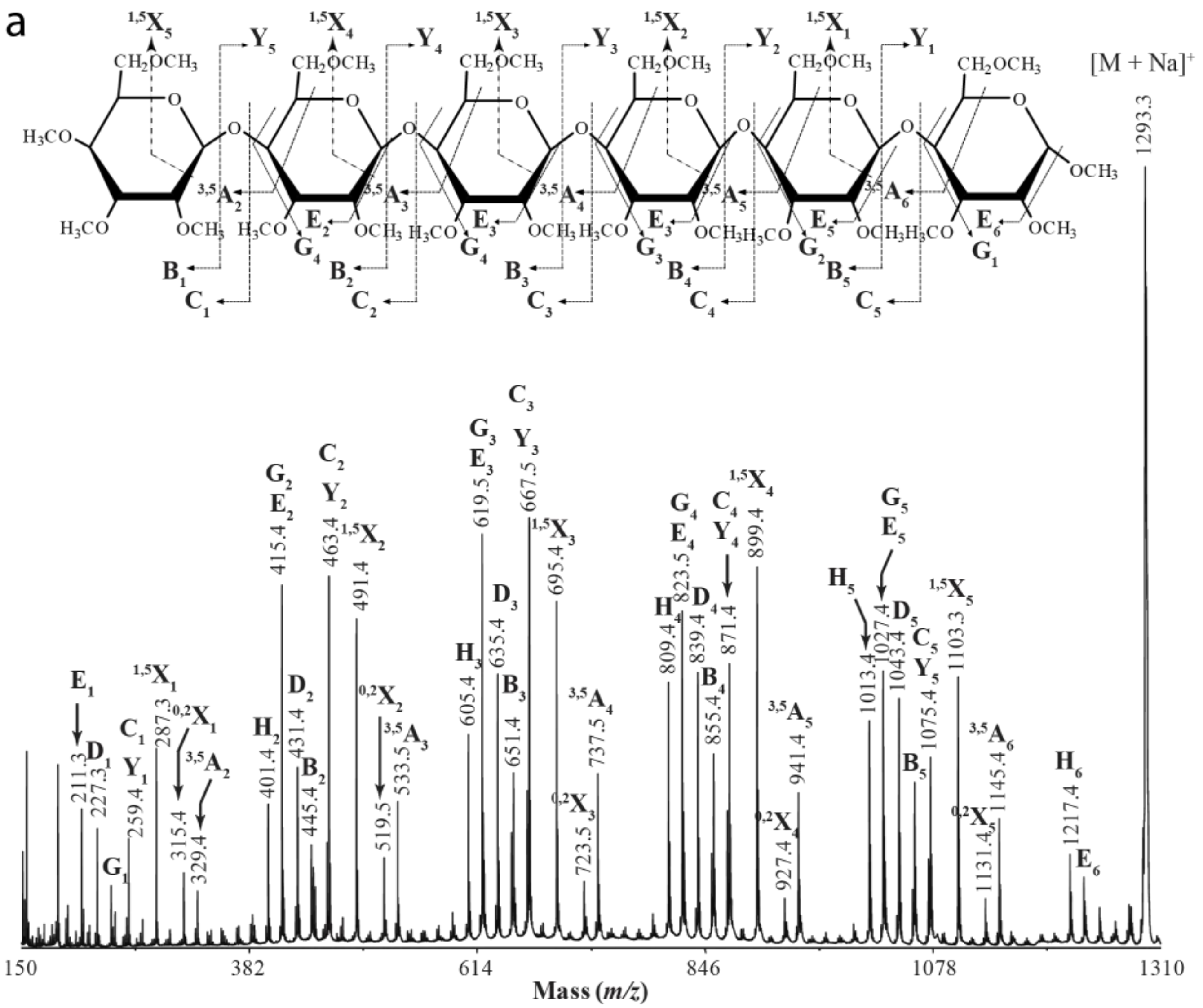

3b)

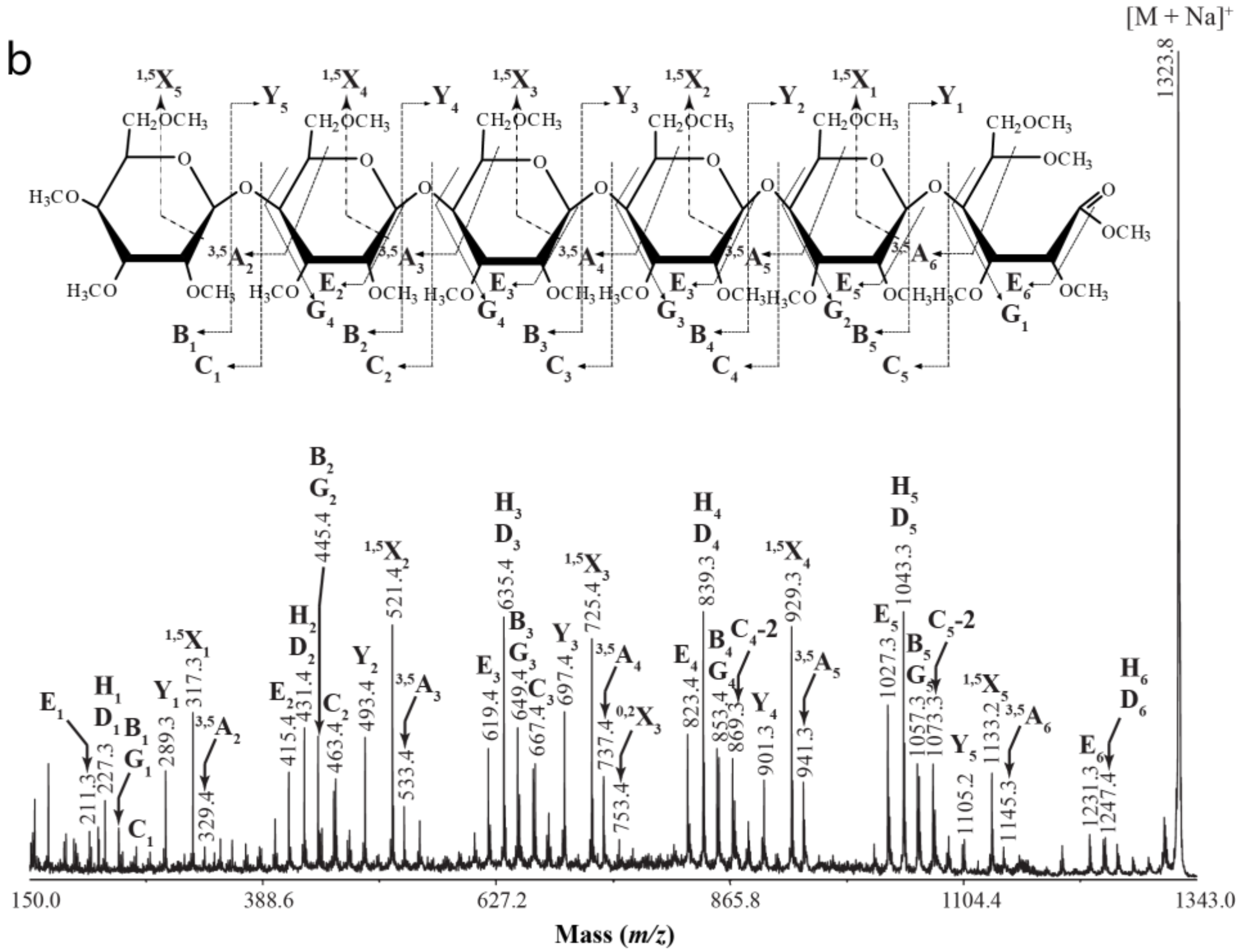

3c)

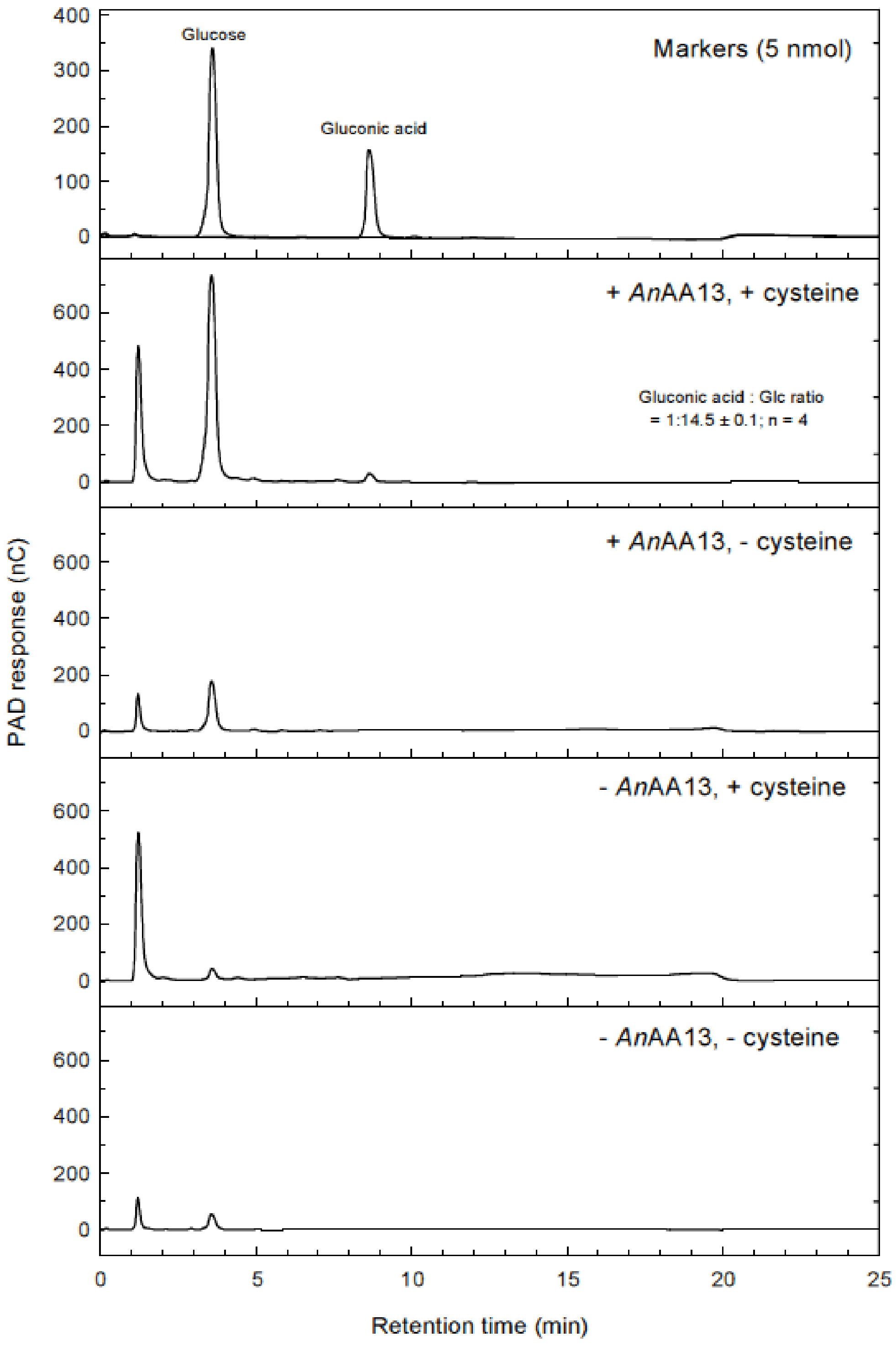
Markers (5 nmol)
Glucose
Gluconic acid
+ *An*AA13, + cysteine
Gluconic acid : Glc ratio
= 1:14.5 ± 0.1; n = 4
+ *An*AA13, - cysteine
- *An*AA13, + cysteine
- *An*AA13, - cysteine
PAD response (nC)
Retention time (min)
0
100
200
300
400
600
5
10
15
20
25

Supplementary Figure 3

The reducing end of the AA13 products is oxidised to gluconic acid. a,b) MALDI-ToF/ToF-MS/MS of *per*-methylated oligosaccharides. Chemical diagrams show the origin of the observed fragmentation ions. A fragmentation spectrum of a maltohexaose standard is shown in a). The fragmentation spectrum of the DP6 product of *An*(AA13) with L-cysteine on retrograded starch is shown in b). The series of ^{1,5}X and Y ions of the An(AA13) product species are 30 Da larger than those of maltohexaose. Since these ions comprise the reducing end of the oligosaccharide, this confirms that the reducing end is modified. Two independent experiments. c) overleaf twice. HPAEC showing generation of gluconic acid by the action of *An*(AA13) on retrograded starch. Ethanol soluble products of *An*(AA13) with cysteine and retrograded starch were hydrolysed by TFA and analysed by HPAEC-PAD. The Gluconic acid:Glc molar ratio is the mean ± SEM. *n*=4 independent experiments.

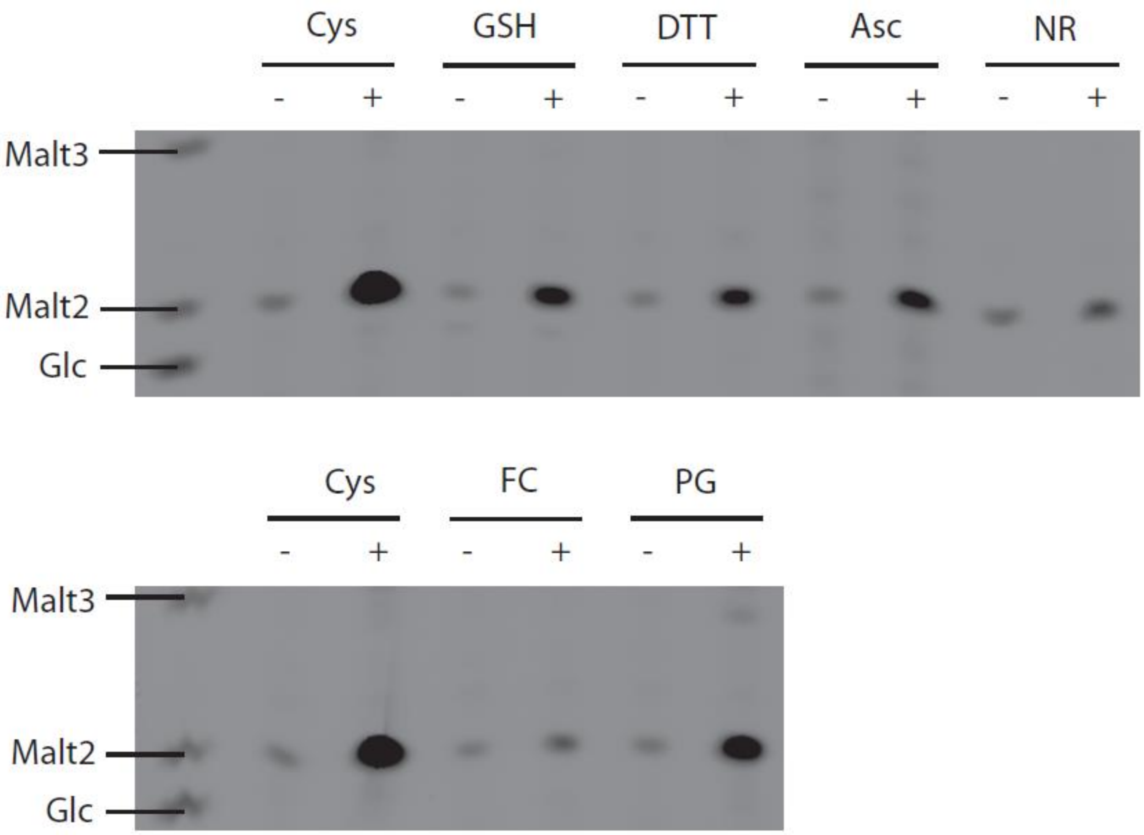

Supplementary Figure 4

PACE gel showing maltose released from retrograded starch over 4 h at 25 °C by action of β-amylase and as boosted by *An*(AA13) in the presence of various reducing co-factors (4 mM). β-amylase stimulation is approximately four-fold higher with Cys compared to Asc as reducing cofactors and twice as high compared to when pyrogallol is used as a co-factor. Under the same conditions the use of cysteine releases 36.8 nmol of maltose, corresponding to 2.5 mol% of available starch. Accordingly, absolute release of maltose with the individual reducing agents can be estimated as follows: GSH, glutathione (10 nmol); DTT, dithiothreitol (10 nmol); Asc, ascorbate (10 nmol); NR, no reductant; FC, ferrocyanide (< 5 nmol); PG, pyrogallol (20 nmol).

+/-, with and without *An*(AA13); Cys, cysteine; GSH, glutathione; DTT, dithiothreitol; Asc, ascorbate; NR, no reductant; FC, ferrocyanide; PG, pyrogallol.

a) b)

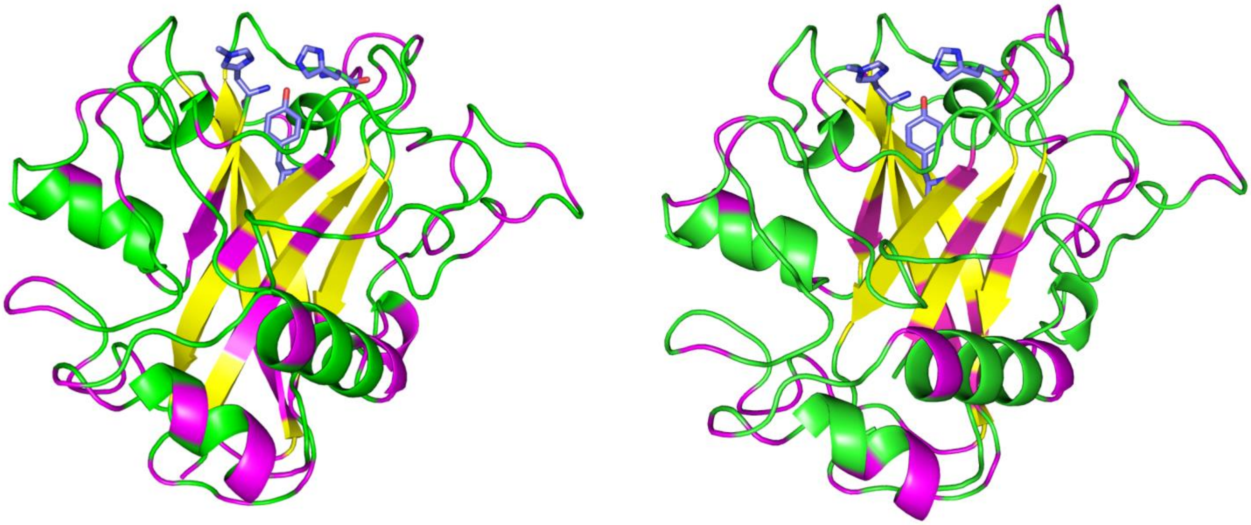

Supplementary Figure 5

Homology models for (a) *An*(AA13) and (b) *Nc*(AA13) based on the *Ao*(AA13) structure. Helices and loops are colored in green, while β-strands are colored in yellow. The active site metal binding residues are in purple while residues that differ in model and template are in magenta.

a) b) c)

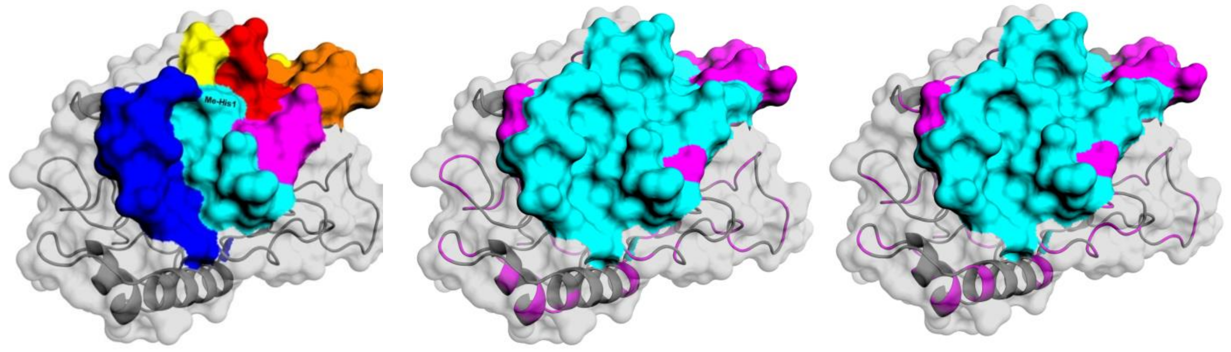

d)

```
An_AA13          HGYLTVPASRTRLGFEAGIDTCPECSILEPVSAWPDLTAAQVGRSGPCGYNARVSVDYNQ  60
Nc_AA13          HGYLTIPFSRTRLGAEAGLDTCPECSILEPVTAWPNVTEAKVGRSGPCGYNARVSIDYNQ  60
Ao_AA13          HGYMYIPSSRTRLGHEAGIDSCPECAILEPVSSWPDLDAAPVGRSGPCGYNARDSIDYNQ  60
                 ***: :* ****** ***:*:****:*****::**::  * ************ *:****
```

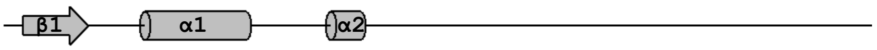


```
An_AA13          PGDYWGNEPVVSYTAGDVVEVQWCVDHNGDHGGMFTYGICQNQTLVDLFLTPGYLPTNEE 120
Nc_AA13          PATNWGNSPVVTYTAGDTVDVQWCVDHNGDHGGMFSYRICQDQELVNKFLTPGYLPTEAE 120
Ao_AA13          PTTNWGSDAVQSYSPGEEIEVQWCVDHNGDHGGMFTYRICQDQSIVDKFLDPSYLPTNDE 120
                 *   **...* :*:.*: ::***************:* ***:* :*: ** *.****: *
```

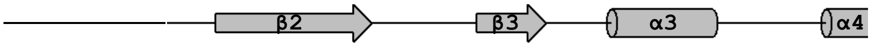


```
An_AA13          KQAAEDCFLEGELSCLHVPGQTCNYNPDCSAGEPCYQNDWFTCNAFQADNNRACQGVDGA 180
Nc_AA13          KQAAEDCFEKGTLPCTDVNGQSCDFSPDCQQGQACWRNDWFTCNAFQADSRRGCQGVDNA 180
Ao_AA13          KQAAEDCFDAGLLPCTDVSGQECGYSADCTEGEACWRNDWFTCNGFEASDRPKCQGVDNA 180
                 ********  * *.* .* ** *.:..**  *:.*::*******.*:*...   *****.*
```

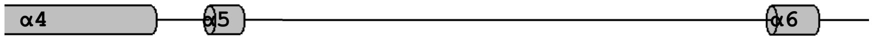


```
An_AA13          ALNSCMTTIAGGYTVTKKIKIPDYSSSHTLLRFRWNSFQTAQVYLHCADIAIV------- 233
Nc_AA13          ALGSCFTTIAGGYTVTKKIKIPNYISGHTLLSFRWNSFQTAQVYLSCADIAIV------- 233
Ao_AA13          ELNSCYTSIAGGYTVTKKVKLPEYTSNHTLISFKWNSFQTGQIYLSCADIAIQ------- 233
                  *.** *:**********:*:*:* *.***: *:******.*:** ******
```

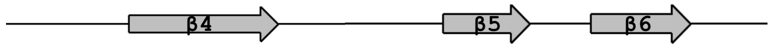

Supplementary Figure 6 Structural elements contributing to AA13 putative substrate binding groove. a) Colored version of surface groove across the active site, showing the distinct regions contributing to it: N-terminus, end of α2 and following residues (1, 25-31) is shown in cyan; part of the loop region preceding β2 (42-45 and 49-55) in blue; loop between β2 and β3 (85-92) in red; part of the long loop region between β3 and β4 including α6 (166-176) in orange; loop region immediately preceding β4 and beginning of β4 (178-180, 189, 193) in yellow; loop region between β5 and β6 (219-224) in magenta. The N-methylated histidine of the active site is indicated (Me-His1). b) and c) show the same view of the surface groove, but with the groove region highlighted in cyan and residues differing between *An*(AA13) and *Ao*(AA13) (b) or *Nc*(AA13) and *Ao*(AA13) (c) in magenta. Differences are localized at the periphery of the

substrate binding groove. d) overleaf. Sequence alignment of catalytic domains of *An*(AA13) and *Nc*(AA13) with *Ao*(AA13) (ClustalX). Identical residues in the three sequences are indicated by asterisks below the alignment. Regions contributing to the putative substrate binding groove are colored as in a). Secondary structure elements as observed in the structure of *Ao*(AA13) are shown below the alignment.

7a)

```
                  β1              α1                           α2
AoAA13        1 HGYMYIP......SSRTRLGHEAG...................IDSCPEC
3ZUD_AA9      1 HGFVQNIVIDGKNYGGYLVNQYPY....MSNP..PEVIAWSTTA...TDL
4MAI_AA11     1 HMMMAQP..............................VPYGKDT...LNN
2YOY_AA10     1 HGYIKEP......VSRAYMGALEKQTMGWTAAAQK.....YGSVIDNPQS

AoAA13       26 AILEPVSSWPDLDAAPVGRSGPCGYNA...RDSIDYNQ....PTTNWGSD
3ZUD_AA9     42 GFVDG.........TGYQTPDIICHRG.........AKP...G.....AL
4MAI_AA11    18 SPLAA.........DG...SDFPCKLRSN.......TYQ...V.....TE
2YOY_AA10    40 VEGPK.....GFPAAGPPDGRIASANGGSGQIDFGLDKQTADH.....WV

                         β2              β3         α3
AoAA13       69 AVQSYSPGEEIEVQWCVDHNG.DHGGMFTYRICQDQSIV.DKFLDPSYLP
3ZUD_AA9     66 TAPVSP..GGTVELQWTPW.PDSHHGPVINYLAPCNGDCSTVD.......
4MAI_AA11    41 ENTAAI..GQSMPLSFIGS.AVHGGGSCQVSLTTD......RE.......
2YOY_AA10    80 KQNIRG..GFNTFTWHYTA.P.HATSKWHYYITKKNWNP.NKP.......

                        α4         α5
AoAA13      117 TNDEKQAA.EDCFDAGLLPCTDVSGQECGYSADCTEGEACWRNDWFT...
3ZUD_AA9    106 ......KT.QLEF..FKIA........................ESGLIN
4MAI_AA11    75 ......PTKDSKW..IVIK........................SIE...
2YOY_AA10   118 ......LS.RDEF..ELIG........................TVN...

                                   α6
AoAA13      163 .......CNGFEASDRPKCQG...............VDNAELNSCYTSIA
3ZUD_AA9    122 DDNPPGIWAS......DNLIA...............AN............
4MAI_AA11    89 .........G......GCPANVDGNLSGGPTSTGASK.............
2YOY_AA10   131 .......HDG......SKADT...............N............

                     β4                 β5                β6
AoAA13      191 GGYTVTKKVKLPE.YTSNHTLISFKWNSFQT......GQIYLSCADIAIQ
3ZUD_AA9    139 ....NSWTVTIPTTIAPGNYVLRHEIIALHSAQNQDGAQNYPQCINLQVT
4MAI_AA11   111 ......FTYTIPEGIEPGKYTLAWTWFNRIG.....NREMYMNCAPLTVT
2YOY_AA10   140 ....LTHKIFVPT.DRSGYHIILGVWDVADT......SNAFYNVIDVNLT

AoAA13
3ZUD_AA9    185 GGGSDNPAGTLGTALYHDTDPGILINIYQKLSSYIIPGPPLYTG
4MAI_AA11   150 GSSSKRDEVPKEKTVEKRSANFPPMFVANVNGCTTKEGVDIRFPNPGSIV
2YOY_AA10

AoAA13
3ZUD_AA9
4MAI_AA11   200 EYAGDKSNLAAEGSQAC
2YOY_AA10
```

7b)

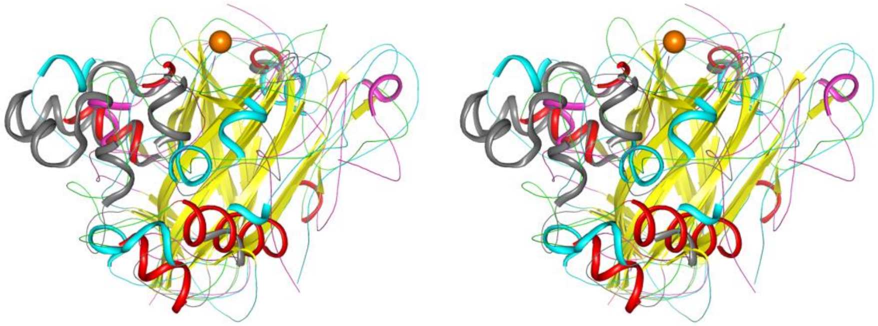

Supplementary Figure 7

Overall structural comparison of LPMO families a) Structure-based sequence alignment (STRAP) of the same enzymes, with strands indicated in yellow and helices in orange. Residues around the catalytic copper are in red font. b) Stereo view of overall overlay of representatives of AA9, AA10, AA11 and AA13. For clarity, all strands are represented in yellow for all structures. *Ao*(AA13) is colored with helices in red and loop regions in green. AA9 (PDB code 3zud) is in cyan, AA10 (PDB code 2yoy) is in dark grey and AA11 (PDB code 4mai) is in magenta. The active site copper is shown as an orange sphere. The figure clearly shows, that beyond the core β-structure, conservation of loops and additional secondary structure elements is low across these AA families.

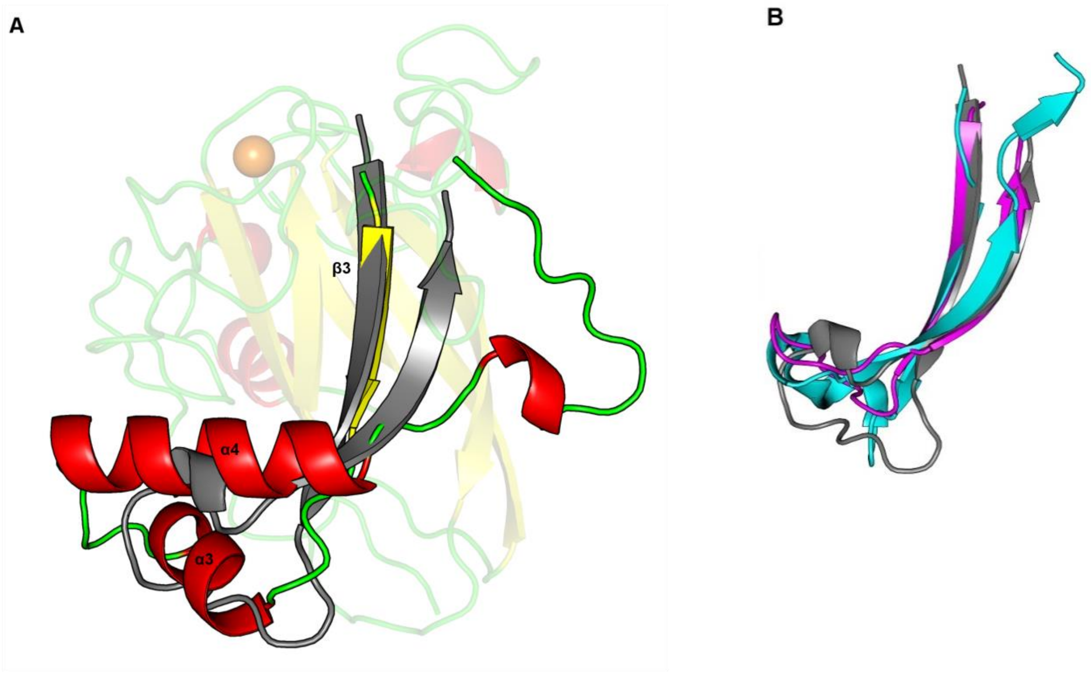

Supplementary Figure 8

'Unique' β3-α3-α4 region in AA13, a) Cartoon representation of *Ao*(AA13) with marked β3-α3-α4 region. The copper ion is shown as an orange sphere. The equivalent region (in grey) of AA10 (PDB 2yoy) is also shown, representing the other LPMO families for which structures are known. None of the other structures (AA9-AA11) has comparably extended α-regions. b) The equivalent regions of AA9 (PDB 3zud), AA10 (PDB 2yoy), AA11 (PDB 4mai) are superimposed and shown in cyan, grey, and magenta, respectively, showing high structural similarity.

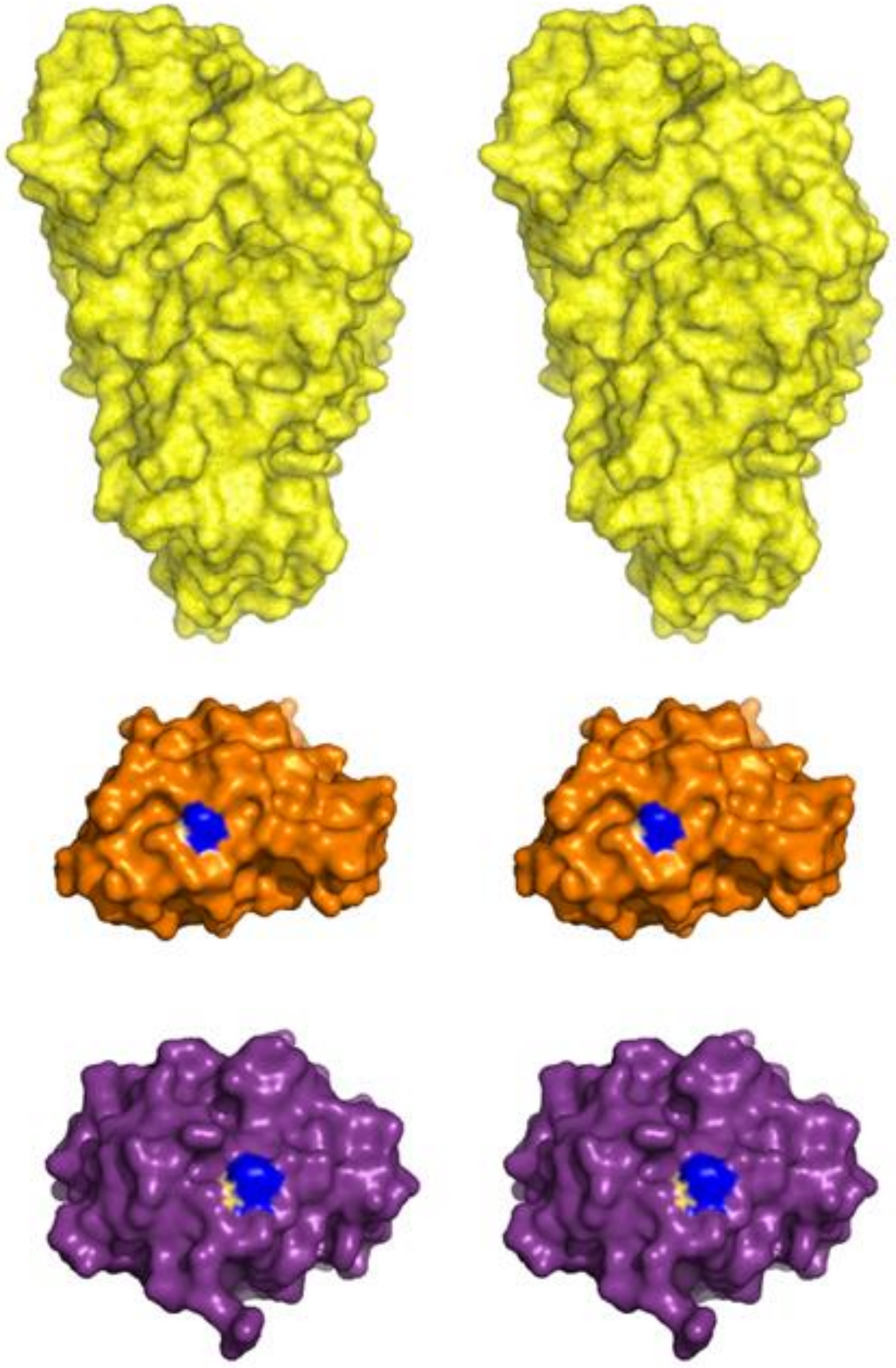

Supplementary figure 9.
Stereoviews of the substrate binding grooves of a *Bacillus* GH13 amylase (PDB code 1wpc, in yellow) and representatives of families AA10 from *Bacillus amyloliquefaciens* (PDB code 2yoy, in orange) and AA11 from *Aspergillus oryzae* (4mai, in purple). The terminal histidine is shown in blue, copper ion shown in light orange.

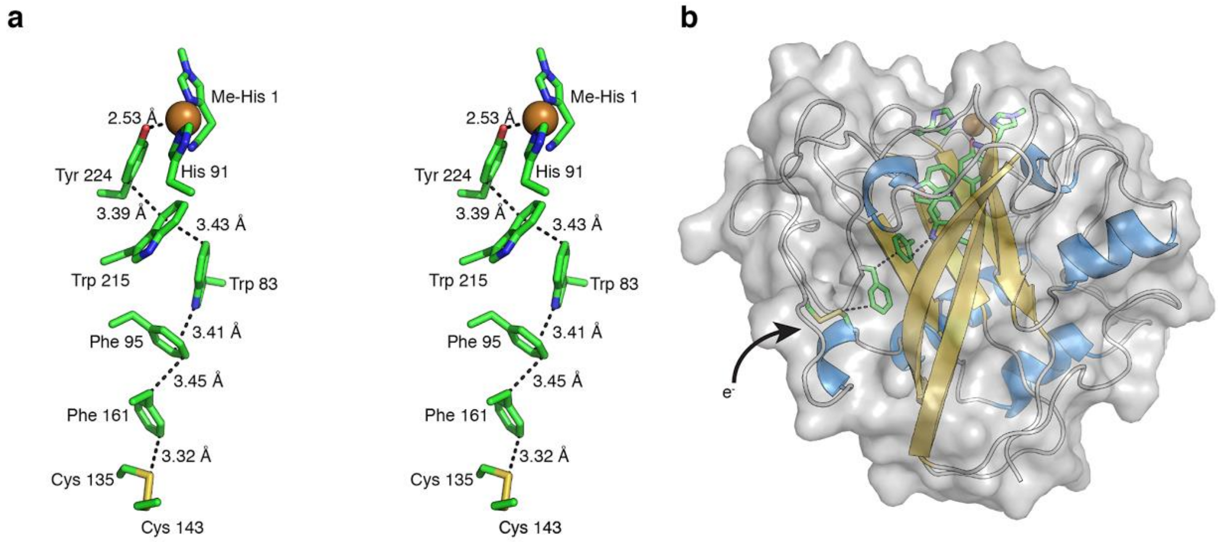

Supplementary Figure 10

a) Stereoview of putative electron transfer pathway from active site tyrosine (Y224) to distal surface-exposed cysteine, generated from *Ao*(AA13) structure. All residues are fully conserved in AA13 sequences save for the occasional substitution of a phenylalanine for a tyrosine, or tryptophan for phenylalanine or lysine. Minimum aromatic C…C or aromatic C…S distances between each adjacent pairs: Y224…W215 3.45, W215…W83 3.39, W83…F95 3.38, F95…F161 3.71, F161…C135 3.48 Å b) overall structure of Ao(AA13) showing placement of putative electron transfer pathway.

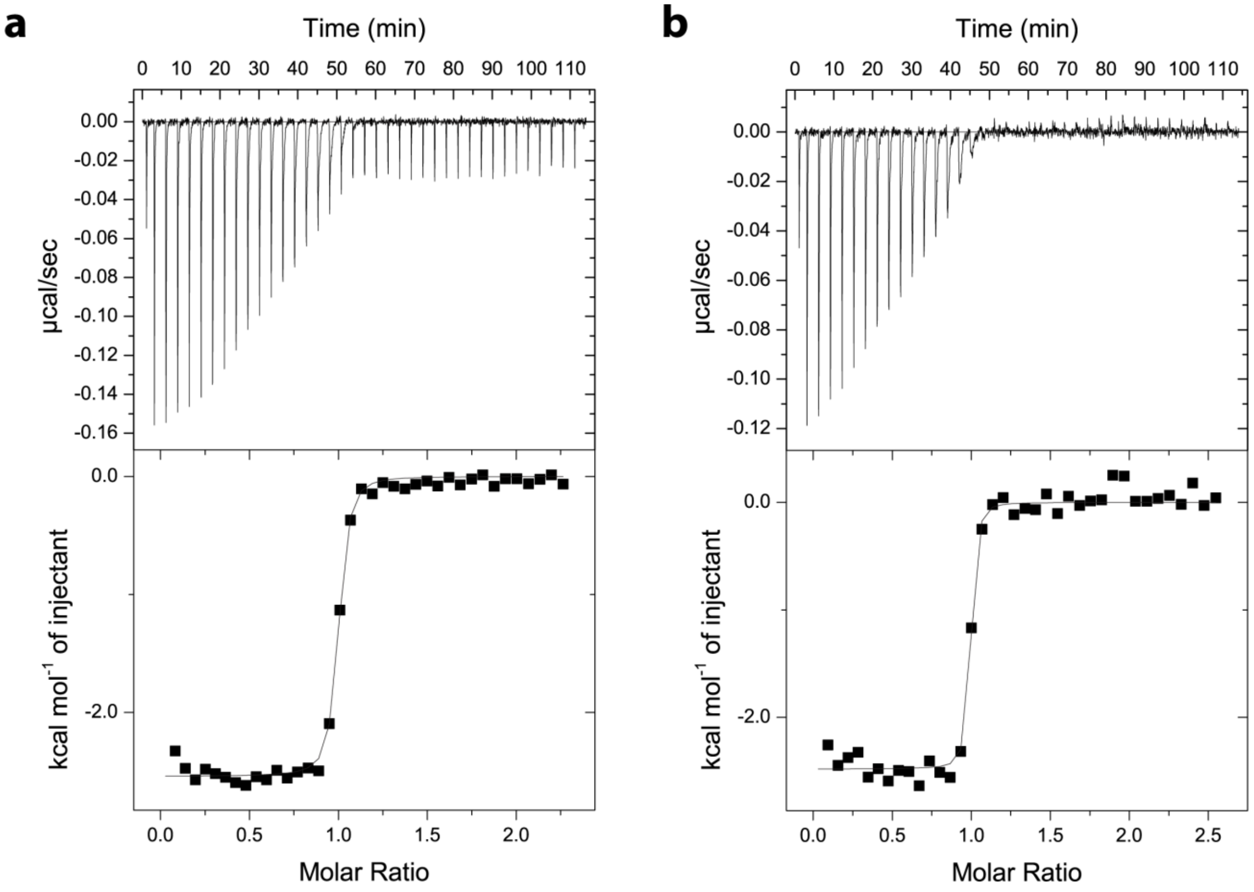

Supplementary Figure 11

ITC thermograms of copper binding. Thermograms for a) *An*(AA13) and b) *Ao*(AA13) showing tight binding of copper to both enzymes as observed for other LPMOs.

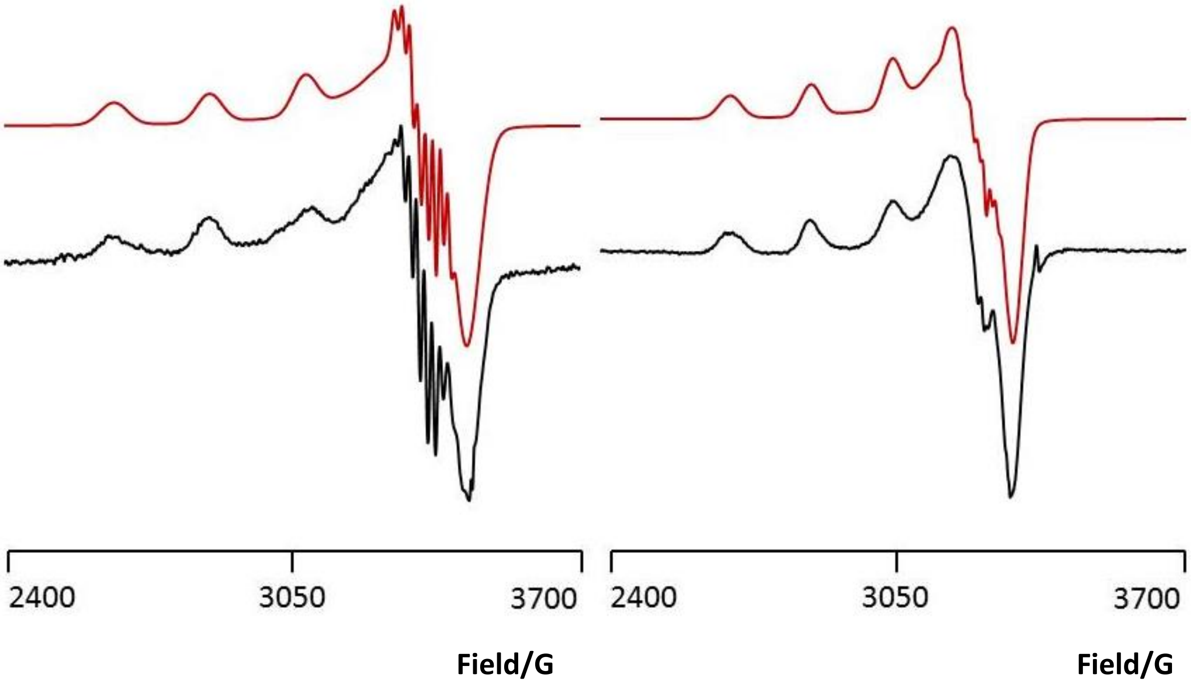

Supplementary Figure 12

X band EPR spectra (frequency ~9.3 GHz, black experiment, red simulation) at 155 K of a) Cu(II)-*An*(AA13) at pH 5, 10% *v/v* glycerol. Cu(II)-*Ao*(AA13) has identical simulation parameters. b) Cu(II)-*An*(AA13) at pH 8.5 with 100 equivalents of azide, 10% *v/v* glycerol.

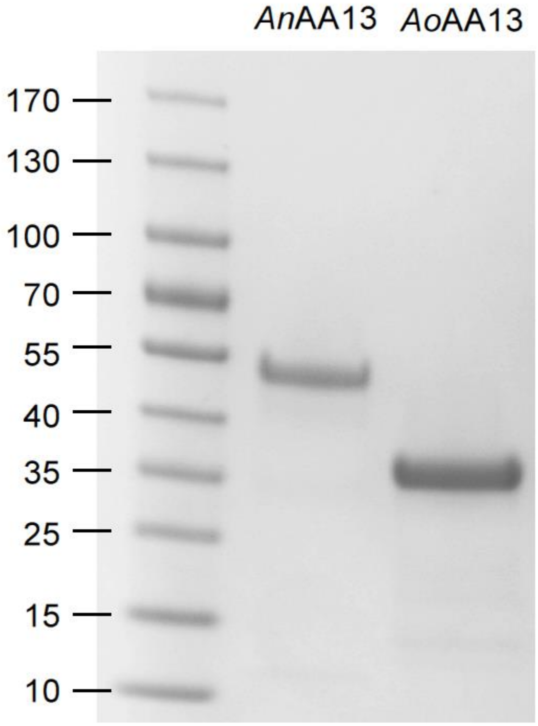

Supplementary Figure 13.
SDS-PAGE gel of *An*(AA13) and *Ao*(AA13), lane 1 molecular weight markers, lane 2 *An*(AA13), lane 3 *Ao*(AA13).

Supplementary Tables

Supplementary Table 1. Table of RMSDs (in Å) between all atoms of active site residues of AA13 and other LPMO containing families.

**AA9**	**2VTC**<br>(32 atoms)<br>H1-H89-Y176	**3EJA**<br>(32 atoms)<br>H1-H68-Y153	**3ZUD**<br>(33 atoms)<br>H1-H86-Y175	**4EIR**<br>(33 atoms)<br>H1-H84-Y168	**4EIS**<br>(33 atoms)<br>H1-H82-Y171	**4B5Q**<br>(32 atoms)<br>H1-H76-Y160
***Ao*(AA13)**<br>(33 atoms)<br>H1-H91-Y224	32 atoms<br>0.96	32 atoms<br>1.04	33 atoms<br>0.73	33 atoms<br>1.05	33 atoms<br>1.04	32 atoms<br>0.53

**AA10**	**2BEM**<br>(31 atoms)<br>H28-H114-F187	**4ALS**<br>(31 atoms)<br>H29-H114-F185	**3UAM**<br>(31 atoms)<br>H19-H122-F205	**2YOY**<br>(31 atoms)<br>H28-H125-F196	**2XWX**<br>(31 atoms)<br>H24-H121-F193	**4OY6**<br>(32 atoms)<br>H43-H150-Y219	**4OY7**<br>(31 atoms)<br>H35-H144-F219	**4GBO**<br>(32 atoms)<br>H37-H144-Y213
***Ao*(AA13)**<br>(33 atoms)<br>H1-H91-Y224	31 atoms<br>0.58	31 atoms<br>0.52	31 atoms<br>1.72	31 atoms<br>0.53	31 atoms<br>1.01	32 atoms<br>0.87	31 atoms<br>0.61	32 atoms<br>1.09

**AA11**	**4MAI**<br>(32 atoms)<br>H1-H60-Y140
***Ao*(AA13)**<br>(33 atoms)<br>H1-H91-Y224	32 atoms<br>0.60

Supplementary Table 2. EPR spin Hamiltonian parameters for Cu-*An*(AA13)

Sample	g_x	g_y	g_z	A_x	A_y	A_z	Strains
**Cu-*An*(AA13) pH 5**<br><br>Line widths 0.6, 0.5	2.0473	2.0767	2.259	27 G (77.78 MHz)<br><br>N shf, 37, 38, 38, 36, 37, 36 MHz	33 G (97.6 MHz)	162 G (513 MHz)	A/G 100, 130, 10<br><br>g 0.008, 0.009, 0.025
**Cu-*An*(AA13) pH 8 + excess azide**<br><br>Line widths 0.6, 0.5	2.038	2.075	2.235	15.7 G (45 MHz)<br><br>N shf 36, 38 38, 35 35, 35	24.1 G (70 MHz)	172.5 (540 MHz)	A/G 40, 160 20<br><br>g 0.008 0.006 0.02

Supplementary Table 3. Known spin Hamiltonian parameters for LPMOs with CAZy classification

CAZy classification (substrate)	g_x	g_y	g_z	A_z /G	Deviation from axial type 2	Source
met*-Cu-AA9 *T. aurantiacus **(cellulose)**	2.06	2.06	2.27	153	None	Ref[1]
met*-Cu-AA10 *B. amyloliquefaciens **(chitin)**	2.05	2.08	2.23	125	reduced A_z value<br>some rhombicity in $g_{x,y}$	Ref[2]
met*-Cu-AA11 *A. oryzae **(chitin)**	2.04	2.10	2.28	157	rhombicity in $g_{x,y}$,	Ref[3]
met*-Cu-AA13 *A. nidulans **(starch)**	2.05	2.08	2.26	162	some rhombicity in $g_{x,y}$<br>resolution of nitrogen SHF	

Supplementary Discussion

Structural features and comparison with other LPMO families

The structure of *Ao*(AA13) consists of six β-strands (β1-β6), arranged as two anti-parallel β-sheets (formed by strands β1-β2-β4 and β3-β5-β6) forming an immunoglobulin-like β-sandwich domain, with the β-strands intercalated by loops occasionally containing helical stretches. The AA13 structure lacks one of the core β-strands, between β3 and β4 in the current numbering, found in the other three LPMO families (AA9, AA10 and AA11), which is instead replaced by a unique motif composed by two helices and a long loop region (Supplementary Fig. 7 and Supplementary Fig. 8). This motif starts with β3, which is relatively short compared to the equivalent β-strand of AA9/AA10/AA11, leading to two α-helices separated by a short loop (α3-α4).

The *Ao*(AA13) structure was structurally aligned with available AA9, AA10 and AA11 structures. For AA9 family members (PDB codes 3ZUD, 3EJA, 2VTC, 4EIR, 4EIS, 4B5Q) 114-134 residues could be structurally aligned to Ao(AA12) with Cα rmsd between 2.5 -3.0 Å and sequence identity between 10.4% and 16.7%. For AA11 (PDB code 4MAI) 123 residues could be aligned with Cα 2.0 Å rmsd and 13.8% sequence identity. The overall structural similarities with family AA10 are slightly higher. For AA10 family members (PDB codes 4ALS, 3UAM, 2YOY, 2XWX, 2BEM) 137-150 residues could be aligned with Cα rmsd between 2.2 -2.3 Å and sequence identity between 13.1% and 17.3%.

Substrate binding surface

A shallow groove runs across the active site. Supplementary Fig. 6 shows in more detail which part of the groove is contributed by which region. These regions are also color coded on a sequence alignment of *Ao*(AA13), *An*(AA13) and *Nc*(AA13). This groove is shallower than those often observed for α-amylases but deeper than any surface contour seen in the known structures of AA9, AA10 and AA11 LPMOs (see stereoviews in figure 5 and Supplementary figure 9), leading us to speculate that AA13 LPMOs can bind effectively glucan chains partly embedded in crystalline/amorphous regions. Simple docking models (not shown) indicate that a single glucan chain is spatially accommodated within the surface groove of *Ao*(AA13), although given the complexity of the surface of retrograded starch such modelling cannot account for the wide range of possible surface profiles that will be present on the substrate.

References

1 Quinlan, R. J. *et al.* Insights into the oxidative degradation of cellulose by a copper metalloenzyme that exploits biomass components. *Proceedings of the National Academy of Sciences* **108**, 15079-15084 (2011).

2 Glyn R. Hemsworth, E. J. T., Robbert Q. Kim, Rebecca C. Gregory, Sally J. Lewis, Johan P. Turkenburg, Alison J. Parkin, Gideon J. Davies and Paul H. Walton. The copper active site of CBM33 polysaccharide oxygenases. *J Am Chem Soc* **135**, 6069-6077 (2013).

3 Hemsworth, G. R., Henrissat, B., Davies, G. J. & Walton, P. H. Discovery of a new family of lytic polysaccharide mono-oxygenases. *Nature Chemical Biology* **10**, 122-126 (2014).
